# Global Epidemiology and Seasonality of Human Seasonal Coronaviruses: A Systematic Review

**DOI:** 10.1093/ofid/ofae418

**Published:** 2024-07-18

**Authors:** Rory Wilson, Dory Kovacs, Mairi Crosby, Antonia Ho

**Affiliations:** Department of Global Health and Population, Harvard T.H. Chan School of Public Health, Boston, Massachusetts, USA; College of Medical, Veterinary, and Life Sciences, University of Glasgow, Glasgow, UK; College of Medical, Veterinary, and Life Sciences, University of Glasgow, Glasgow, UK; Medical Research Council-University of Glasgow Centre for Virus Research, University of Glasgow, Glasgow, UK

**Keywords:** acute respiratory illness, epidemiology, human seasonal coronavirus, seasonality, systematic review

## Abstract

**Background:**

We characterized the global epidemiology and seasonality of human coronaviruses (HCoVs) OC43, NL63, 229E, and HKU1.

**Methods:**

In this systematic review, we searched MEDLINE, EMBASE, Web of Science, SCOPUS, CINAHL, and backward citations for studies published until 1 September 2023. We included studies with ≥12 months of consecutive data and tested for ≥1 HCoV species. Case reports, review articles, animal studies, studies focusing on SARS-CoV-1, SARS-CoV-2, and/or Middle East respiratory syndrome, and those including <100 cases were excluded. Study quality and risk of bias were assessed using Joanna Briggs Institute Critical Appraisal Checklist tools. We reported the prevalence of all HCoVs and individual species. Seasonality was reported for studies that included ≥100 HCoVs annually. This study is registered with PROSPERO, CRD42022330902.

**Results:**

A total of 201 studies (1 819 320 samples) from 68 countries were included. A high proportion were from China (19.4%; n = 39), whereas the Southern Hemisphere was underrepresented. Most were case series (77.1%, n = 155) with samples from secondary care (74.1%, n = 149). Seventeen (8.5%) studies included asymptomatic controls, whereas 76 (37.8%) reported results for all 4 HCoV species. Overall, OC43 was the most prevalent HCoV. Median test positivity of OC43 and NL63 was higher in children, and 229E and HKU1 in adults. Among 18 studies that described seasonality (17 from the Northern Hemisphere), circulation of all HCoVs mostly peaked during cold months.

**Conclusions:**

In our comprehensive review, few studies reported the prevalence of individual HCoVs or seasonality. Further research on the burden and circulation of HCoVs is needed, particularly from Africa, South Asia, and Central/South America.

Human coronaviruses (HCoV) are common respiratory pathogens named after their crown-like structure [1]. Among the 7 coronaviruses that infect humans, severe acute respiratory syndrome coronavirus 1 (SARS-CoV-1) and Middle East respiratory syndrome (MERS-CoV) have caused severe epidemics of respiratory illness, whereas SARS-CoV-2, which emerged in 2019, continues to cause significant waves of infection worldwide. As of 15 June 2024, there have been more than 775 million confirmed cases of SARS-CoV-2 infection and approximately 7 million associated deaths worldwide [2]. The high disease burden and mortality mean that the potential periodic oscillation of this virus is crucial to predict so health care systems can better anticipate resource allocation.

Four species of HCoVs are endemic and are known to circulate seasonally, including 2 alphacoronaviruses (NL63 and 229E) and 2 betacoronaviruses (OC43 and HKU1) [3]. They typically cause mild respiratory illness in winter months in the Northern Hemisphere [4] but can cause severe respiratory presentations, particularly in immunocompromised individuals [5, 6]. HCoV circulation generally peaks in winter months in the Northern Hemisphere, but less is known about the circulation patterns in the Southern Hemisphere. Moreover, existing reviews have only characterized the seasonality and prevalence of detection aggregated for all HCoVs and have not evaluated studies that included contemporaneous controls to ascertain the clinical significance of HCoV detection. Studying the epidemiology and seasonality of individual seasonal coronavirus species, particularly betacoronaviruses, may provide insight into the future trajectory of SARS-CoV-2 and its potential of becoming an endemic virus [7]. Moreover, prior exposure to seasonal coronaviruses may offer a degree of protection against severe COVID-19 [8, 9].

This review aimed to (1) characterize the prevalence and incidence of human seasonal coronavirus species (OC43, NL63, 229E, and HKU1) in acute respiratory illness (ARI), as well as asymptomatic controls, in children and adults, and (2) describe the seasonal patterns of individual HCoV species by geographic region.

## METHODS

### Search Strategy and Selection Criteria

This systematic review was reported in accordance with PRISMA statement [10] and is registered with the International Prospective Register of Systematic Review (PROSPERO; CRD42022330902). We retrieved all English-language research articles reporting the epidemiology of human seasonal coronaviruses through systematic searches of major databases, including MEDLINE, EMBASE, Web of Science, SCOPUS, and CINAHL, using subject heading terms for studies published up to 1 September 2023. We also manually screened the references of included studies to identify additional studies. Oxford Centre for Evidence-Based Medicine 2011 [11] was used to determine appropriate study designs. Initial search of databases comprised keywords (or appropriate synonyms for different databases): Human coronavirus AND respiratory disease AND epidemiology OR seasonality.

Studies were eligible if they met the following inclusion criteria: prospective or retrospective study containing at least 12 months of consecutive data and tested for 1 or more species of HCoV. We excluded review articles, animal studies, studies that focused on SARS-CoV-1, SARS-CoV-2, and/or MERS, case reports or case series with fewer than 100 cases (likely from reporting bias), and where full text was not available in English. The detailed search strategy is shown in the Supplementary Materials (Appendix 1).

### Data Extraction

Three authors (M. C., D. K., A. H.) independently performed study selection according to the eligibility criteria; each record was screened by 2 reviewers. Three authors (M. C., D. K., R. W.) independently extracted the data from study articles using a prespecified template. From each eligible study, the following variables were extracted as a minimum: title, name of the first author, year of publication, study setting population, single or multisite, inclusion of controls, country/countries, study design, study period, inclusion of a 12-month period, participant and sample size, participant age category (adult vs pediatric, as specified by original study), sex, clinical criteria for testing for HCoV, testing method, HCoV species tested (OC43, NL63, 229E, and HKU1), proportion testing positive of all HCoVs and of individual species, month or season with the highest prevalence or incidence, in addition to HCoV reinfections and coinfections with other respiratory viruses.

### Data Synthesis

For studies that tested for all 4 HCoVs, we reported the median and interquartile range (IQR) of the prevalence of all HCoVs and of individual HCoV species in all participants, and by age category (pediatric vs adults, as specified by original study). Because of the heterogeneity of ARI case definition applied by included studies and lack of specification of testing criteria in some studies, we opted not to summarize prevalence by severity of respiratory illness. Incidence rates, where available, in addition to HCoV reinfection and coinfections with other respiratory viruses were also reviewed. We summarized seasonal patterns in studies that reported prevalence by month and included at least 100 HCoV-positive samples over a 12-month period. Last, in studies that included asymptomatic controls (individuals that reported no ARI symptoms at the time of testing), we compared the prevalence of HCoVs between cases and controls to ascertain the likely clinical relevance of HCoV detection.

### Risk of Bias

Three authors (M. C., D. K., R. W.) assessed the quality and risk of bias of all studies using the Joanna Briggs Institute Critical Appraisal Tools [12], which comprise standardized checklists for case series, cohort, and case-control studies included in this review. Each study was independently appraised by 2 reviewers, and any areas of conflicts were resolved by a fourth author (A. H.). We used the 10 questions (11 for cohort) to systematically evaluate the risk of bias of each study. Items with high risk of bias (answered as “no”) were assigned a score of 1; items that were unclear risk of bias (answered as unclear) were assigned a score of 0.5; and items with low risk of bias (answered as “yes”) were assigned a score of zero. Using a risk of bias scoring system adapted from Park et al [13], reviewers classified studies as being at low risk (0–1.5), medium risk (2–4), or high risk (4.5–10), in the case of cohort studies, high risk ranged from 4.5 to 11.

## RESULTS

### Search Results

**Figure 1.**
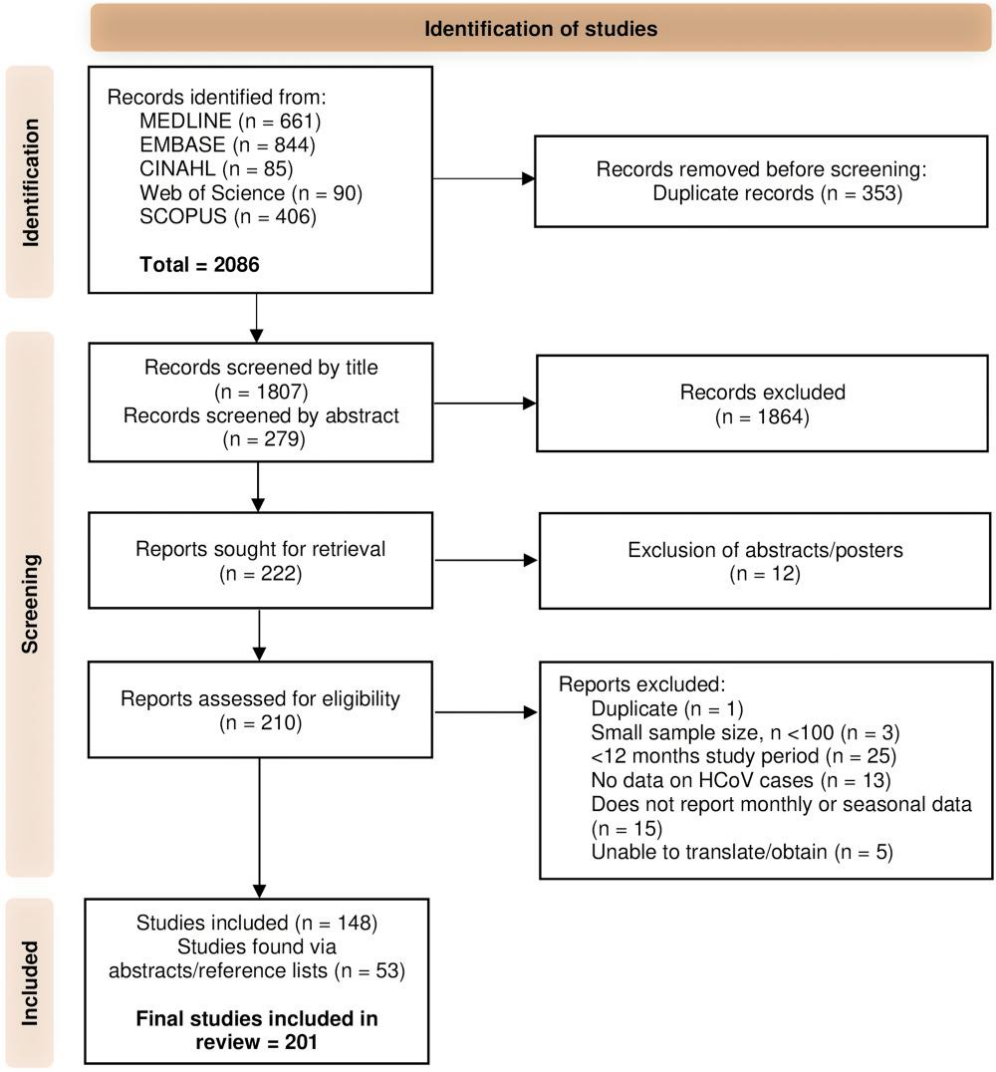
Preferred Reporting Items for Systematic Reviews & Meta-Analyses (PRISMA) flowchart.

Our initial search identified 2086 studies up until 1 September 2023, from which 353 duplicates were removed (Figure 1). A further 1864 studies were removed for reasons, such as focus on a nonendemic coronavirus (MERS-CoV, SARS-CoV-1, and SARS-CoV-2) or lack of mention of human coronavirus(es); animal and case studies were also excluded. After full-text screening and exclusions because of small sample size or insufficient HCoV data or study period, 148 eligible studies met the eligibility criteria. An additional 53 studies were identified through reference lists of included publications (n = 201).

### Characteristics of Included Studies

**Figure 2.**
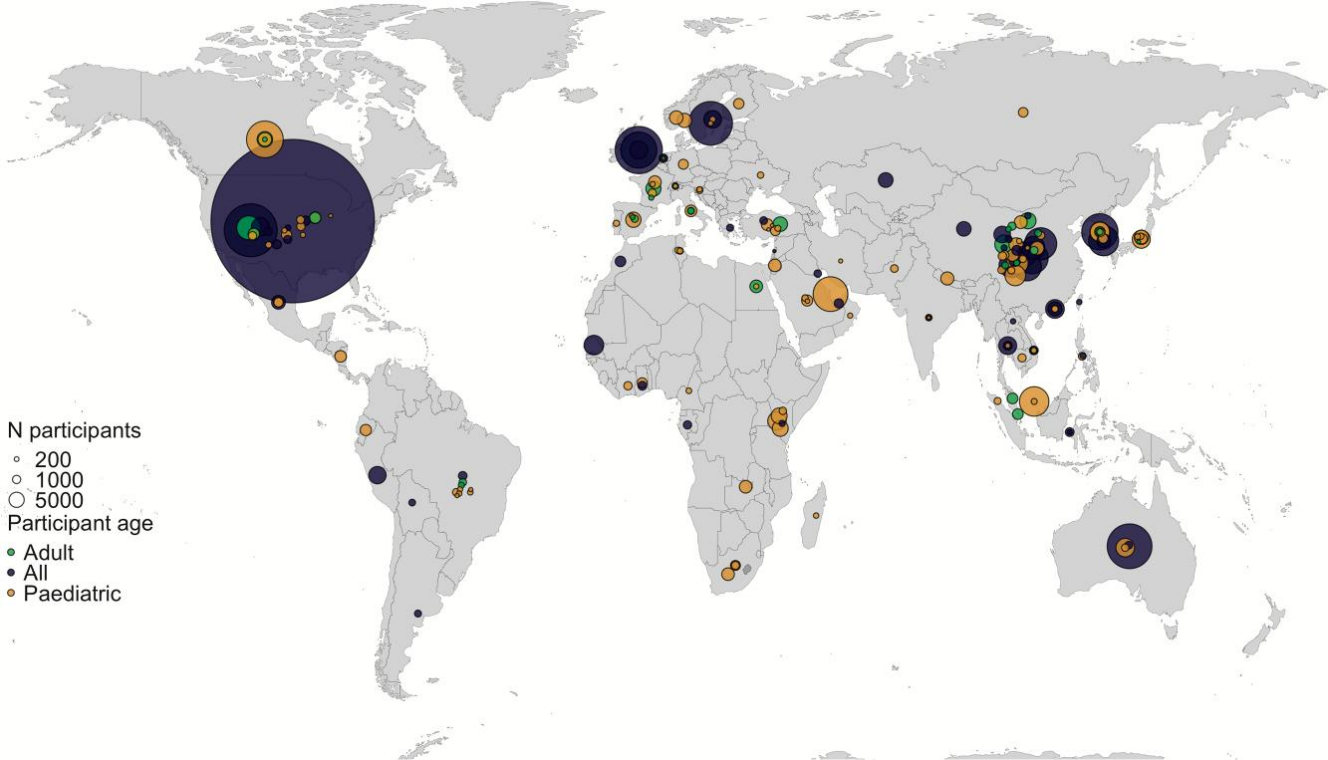
Geographical distribution of included studies, by participant age groups (pediatric, adult, or all) and number of included participants. Studies set in multiple countries are displayed in each country. One study covered Europe and is not shown on the map [23]. If a country reported several studies, the bubbles were jittered to avoid overlap and improve visibility; these do not reflect the exact location of the study within that country.

The included studies were published between 2006 and 2023; most (77.1%, 155/201) were case series, of which 102 were prospective (Supplementary Table 1). Additionally, we included 17 surveillance, 18 prospective cohort (including 6 birth cohorts [14–19]), and 9 case-control studies. Two studies [20, 21] employed 2 study designs. Only 17 (8.5%) studies included control groups (Supplementary Tables 1 and 2), and half (49.8%, n = 100) included an observation period that included multiples of 12 months. Approximately half (51.7%, n = 104) were multisite studies, and several studies included more than 1 health care and/or community settings. Most studies involved HCoV cases and/or controls from secondary care (74.1%, n = 149); the rest included samples obtained in primary (n = 26) and tertiary care (n = 17) settings, as well as in the community (n = 26). One study, also the largest (n = 854 575 HCoV tests), focused on samples obtained from clinics and laboratories as part of passive surveillance in the United States [22]. The 201 studies encompassed data from 68 countries; China had the highest number of studies (n = 38), whereas 5 involved participants from multiple countries [23–27]. Most publications came from the Northern Hemisphere (Figure 2, Supplementary Table 1).

The median study population was 1002 (IQR 411–3831); half of included studies (49.8%, 100/201) had fewer than 1000 participants. ARI was the most frequently used clinical criterion for testing (93/201, 46.3% studies), though case definitions were highly variable. Several studies included multiple symptom criteria, including influenza-like illness (ILI), pneumonia, and severe acute respiratory illness. Forty-two (20.9%) studies included severe cases exclusively (defined as pneumonia, severe acute respiratory illness, or acute lower respiratory tract infection), whereas 98 (49.2%) investigated only mild infections (defined as ARI, ILI, or upper respiratory tract infection). Symptoms of those tested were unspecified for 17 (8.6%) studies. More than half of included studies (53.7%, 108/201) focused on pediatric populations, whereas approximately one third (30.8%, 62/201) included all ages.

All studies used polymerase chain reaction tests to identify individual HCoV species. However, only 125 (62.2%) stated all 4 HCoV species were included in tests, of which 5 studies grouped alpha- and betacoronaviruses [28–32] (Supplementary Table 1). Of the 201 included studies, 109 reported results for OC43 prevalence, 107 for NL63, 106 for 229E, and 81 for HKU1 (Supplementary Table 3).

### Overall Prevalence of Individual HCoVs

Among the 76 (37.8%) studies that reported results of all 4 HCoV species individually, OC43 was the most prevalent in 39 (51.3%, particularly in pediatric studies [66.7%, 21/45]), followed by NL63 (n = 17; 22.4%). Eight (10.5%) studies reported HKU1 to be the most prevalent, and 6 (7.9%) 229E. Six (7.9%) studies reported similar prevalence of 2 or more HCoVs, whereas Hatem et al [33] reported no cases for all HCoVs.

In terms of geographical regions, 8 of 15 studies that reported individual HCoV results in China found OC43 to be the most prevalent. Several African studies that evaluated all 4 HCoV species also found OC43 to be the most prevalent, including 3 from South Africa [24, 34, 35] and 1 from Kenya [36]. Khalifa et al [37] and Berkley et al [20] reported 229E to be the most prevalent in Tunisia and Kenya, respectively, whereas Venter et al [38] most commonly found NL63 among South African children.

Unexpectedly high prevalence of HCoVs may have been reported during outbreaks [39], if sample size is small [40], or if the study period is 1 year only [41].

### Prevalence of Individual HCoVs by Age

**Figure 3.**
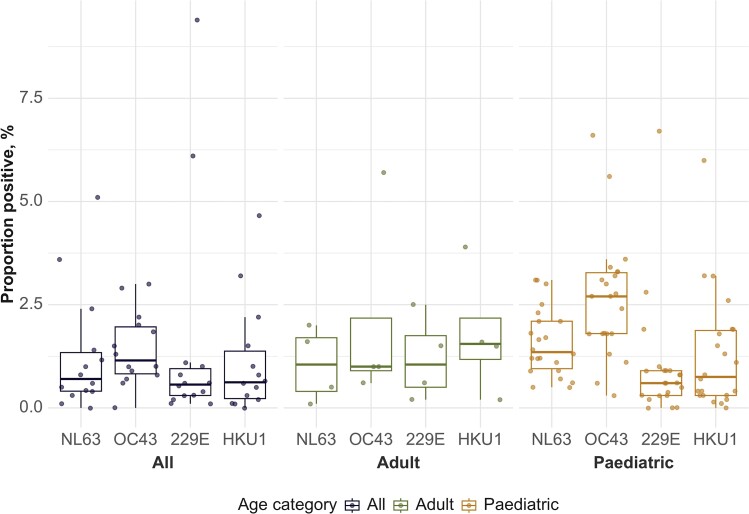
Reported prevalence of human coronavirus species by age group, restricted to studies that included complete 12-month periods. Blue represents values from studies that included participants of all ages, green represents studies including adults only, and orange pediatric populations only. Box plots show median (thick horizontal line and 25th and 75th percentiles [hinges]). Whiskers represent 1.5× interquartile range. Each dot represents a test-positivity value reported by an individual study.

Overall, the prevalence of HCoVs varied by species between children and adults. Among studies that tested for all 4 HCoVs, the overall prevalence of all HCoV species in samples ranged from 1.0% to 9.7% (median, 5.9%). Among studies that reported HCoV prevalence specifically for children or adults, the median prevalence (and IQR) of all HCoVs except 229E was higher in children. When restricted to studies reporting for multiples of 12 months only (to avoid seasonality bias), the median prevalence of OC43 and NL63 was higher in children than in adults; OC43: 2.7% (IQR 1.8%–3.3%) in children versus 1.0% (IQR 0.9%–2.2%) in adults, and NL63: 1.4% (IQR 1.0%–2.1%) in children versus 1.1% (IQR 0.4%–1.7%) in adults, whereas adults had a higher prevalence of HKU1: 1.6% (1.2%–2.2%) in adults versus 0.8% (0.4%–1.9%) in children and 229E: 1.1% (0.5%–1.8%) in adults versus 0.6% (0.3%–0.9%) in children (Figure 3).

### Incidence of HCoV Infections and Reinfections

Of 18 included cohort studies, 7 reported incidence rates of HCoV infection (Supplementary Table 4). Of these, 3 studies reported individual HCoV incidence rates [4, 19, 42]; OC43 had the highest incidence, and 229E had the lowest incidence in 2 pediatric community cohorts [19, 42], whereas a US household study also found OC43 to have the highest incidence in children, particularly in those younger than 5 years of age, whereas 229E and OC43 had the highest incidence among older adults [4].

Reinfections were reported by several cohort studies [21, 43, 44]; in a household study in Kenya between December 2009 and June 2010 [44], repeat infections with NL63, OC43, and 229E were identified in 21%, 5.7%, and 4.0% of household participants, respectively, whereas reinfections were also found to be common in a cohort of Nicaraguan children [43].

### HCoV Coinfections With Other Respiratory Viruses

Coinfections between HCoVs and other respiratory viruses were reported by 57.2% (115/201) of included studies (Supplementary Table 3). However, the number of respiratory viruses tested, and thus coinfections identified, varied substantially across studies.

### Seasonality of HCoVs

Among 21 studies that included at least 100 HCoV-positive samples annually, 18 (9.1%) reported HCoV prevalence by month or season (Table 1). Most (88.9%, 16/18) spanned multiple years of observation (median study period, 5.5 years; IQR 2.5–8.5 years). All but 1 study from Kenya [54] were from the Northern Hemisphere. Of these, only 13 studies reported seasonality for individual HCoV species. In temperate regions, HCoV activity typically peaked during the winter and spring months. In tropical Nicaragua, HCoVs followed no clear seasonal trend, but NL63 peaked in the second half of the year, and, generally, 3 months following a 229E peak [43]. The Kenyan study [54] also observed near year-round circulation of OC43 and NL63, though peak circulation coincided with cool, dry season. The 3 studies from China [31, 47, 56] reported different seasonal peaks, though they were done in different regions over different periods, and none had reported seasonality for individual HCoVs.

**Table 1.** Included Studies That Assessed Seasonality

Study	Country	Time Period	N HCoVs detected	Month or Months^a^ of Peak Circulation Reported			
				OC43 N (%)	NL63 N (%)	229E N (%)	HKU1 N (%)
Al-Romaihi (2020) [**45**]	Qatar	2012–2017	1740	Winter (December-March)			
Choi (2021) [**46**]	South Korea	2015–2019	463	Often 2 peaks annually in winter (November–February) and spring (April–June)	Winter/spring (November–December/February–March)	Winter (November–February), even seasons^b^	NT
Cui (2015) [**47**]	China	2010–2011	155	Spring (April)			
Dyrdak (2021)^d^ [**48**]	Sweden	2009–2020	2130	Winter (December), even seasons	Winter/spring (February), even seasons	Spring (March–April), odd seasons	Winter (December), odd seasons^c^
Frutos (2022) [**43**]	Nicaragua	2011–2016	610	No clear seasonal pattern	Second half of year	No clear seasonal pattern	No clear seasonal pattern
Hawkes (2021) [**49**]	Canada	2005–2017	4657	Winter^f^, even seasons	Winter^f^, even seasons	Winter^f^, odd seasons	Winter^f^, odd seasons
Jo (2022) [**50**]	South Korea	2015–2019	1096	Winter (December–January)	Winter (December–March)/spring (January–May)	Winter (November–February), even seasons	Spring (April 2019 only)
Killerby (2018) [**22**]	USA	2014–2017	39 588	Winter (January–February)	Winter (February–March)	Spring (March–April), odd seasons	Winter (February–March), odd seasons
Kim J-M (2018) **[51**]	South Korea	2013–2015	1537	Winter (December)	Winter (December–February), even seasons	Winter (February, 2013/14 only)	NT
Kim T (2021) [**52**]	South Korea	2018–2020	807	Winter (December)	Winter (November–January)	Winter (January), even seasons	NT
Monto (2020) [**4**]	USA	2010–2018	993	Winter (January)	Winter (January)	Winter (February)	Winter (February)
Nickbakhsh (2016) [**53**]	UK	2005–2013	1339	Winter^f^ (October–March)			
Nickbakhsh (2020) [**3**]	UK	2005–2017	2958	Winter (December–March)	Winter (December–March)	Spring (March–April), even seasons 2006–2012, odd seasons 2013–2017	NT
Nyiro (2018) [**54**]	Kenya	2016	387	Cool, dry season (June). Almost year-round circulation	Cool, dry season (September). Almost year-round circulation	Cool, dry season (September)	NT
Shah (2022) [**55**]	USA	2014–2021	5204 (6.3)	Winter (January–February), until 2020	Winter/spring (January–April)	Spring (March–April), odd seasons 2015–2019	Winter (January–February), even seasons
Shi (2023) [**56**]	China	2021–2022	280 (<0.1)	Summer/autumn (August–September, November)			
Varghese (2018) [**57**]	USA	2013–2014	212 (8.2)	Autumn^e^ (October–December)	Winter^e^ (January–March)	Winter^e^ (January–March)	Winter^e^ (January–March)
Zhang, D (2013) [58]	China	2009–2012	351 (2.5)	Summer (June)			

Abbreviations: HCoVs, human coronaviruses; NT, not tested.

^a^If multiple years included.

^b^When peak HCoV circulation occurred predominantly in years ending in an even number.

^c^When peak HCoV circulation occurred only in years ending in an odd number.

^d^Drydak et al (2021) reported percentages (as HCoV species positive over total HCoV positives) but not counts of positive samples for each HCoV species.

^e^Varghese et al (2018) reported HCoV cases by quarterly year.

^f^Unable to determine month(s) of peak circulation from graph.

Individual HCoVs often displayed distinct seasonality and periodicity. Two large studies that involved routine diagnostic data from Canada [49] (2005–2017) and Sweden [48] (2009–2020) both observed alternating peak winter seasons of the 2 alphacoronaviruses (NL63 and 229E), and similarly for the betacoronaviruses (OC43 and HKU1), usually with OC43 and NL63 circulating in the same season. A Scottish study also identified asynchronous peak circulation of 229E with peak activity of OC43 and NL63, which circulated around the same time [3]. This study also noted that 229E peaked biennially before the 2009 A(H1N1) pandemic but had longer interpeak periods postpandemic.

### Impact of COVID-19 Pandemic on HCoV Epidemiology

Although the study period of 12 studies included the early months/years of the COVID-19 pandemic (1 January 2020 onwards), 2 included the pandemic period only [56, 59], and 6 did not specifically evaluate the impact of the pandemic [60]. Of the 3 studies that spanned both pre- and during-pandemic periods, 2 found substantially lower prevalence of HCoVs in 2020 compared to previous years [21, 61], whereas a US study identified a later peak of NL63 and OC43 in 2021 (in May), and 229E and HKU1 detection remained low [55]. Before 2020, HCoVs peaked in cold months (usually February).

### Studies that Included Controls

Seventeen studies included data from asymptomatic controls, as well as cases (Supplementary Table 2) [20, 23, 24, 35, 38, 62–73]. Seven studies recruited substantially fewer controls than cases [20, 38, 62–66]; 4 had fewer than 200 controls [20, 38, 62, 69]. Moreover, controls were not recruited at the same time as cases in 7 of the studies [20, 62, 63, 65, 66, 68, 73], and timeframe of control recruitment was not mentioned by Venter et al [38], precluding valid case-control comparison in these studies because any observed differences in the prevalence of HCoVs may be due to differing seasonality rather than varying contribution to illness severity.

Although 2 of the US studies [65, 66] tested for all 4 HCoV species, they did not report species-specific HCoV prevalence and did not make any comparison by case-control status. Of 6 studies that enrolled contemporaneous controls, included a sufficient number of controls for statistical comparison, and reported prevalence of individual HCoVs [24, 63, 64, 67, 72], Owusu et al [67] found that 229E and OC43 were associated with upper respiratory tract infection in those aged >10 years. A case-control study nested within a South African birth cohort found that OC43 was associated with lower respiratory tract infection in the first year of life [35]. A Norwegian study demonstrated that a higher HCoV genomic load (defined as cycle threshold < 28 on polymerase chain reaction analysis) was independently associated with respiratory tract infection [64]. The other 3 studies found either no difference in the prevalence of individual HCoVs between cases and controls or a higher prevalence of HCoV in controls, compared to cases [24, 72, 74].

### Risk of Bias

Of the 201 studies, 169 (84.1%) were at low risk of bias, 26 (12.9%) were at medium risk of bias, and 6 (3.0%) were at high risk of bias (Appendix 2). All studies were included in the analysis.

## DISCUSSION

This systematic review is the first to summarize the seasonality and the contribution of individual HCoV species to acute respiratory illness as well as their presence in asymptomatic controls. The prevalence of HCoV infections varied considerably across studies, but overall, OC43 was most frequently detected in both children and adults. For OC43 and NL63, median test positivity was higher in children than in adults, whereas 229E and HKU1 were more prevalent in adults. Most studies were conducted in the Northern Hemisphere, particularly China and the United States, whereas there was a paucity of studies from Africa, South Asia, and Central/South America. Of few studies that included contemporaneous controls, half found no difference in the prevalence of HCoVs between cases and controls, suggesting that identification of an HCoV may not be the causative agent of the ARI. Although seasonality could rarely be evaluated because of limited sample size, studies consistently showed that peak circulation of HCoVs occurred in winter months in temperate regions. Last, several studies demonstrated attenuated or disturbed HCoV circulation during the COVID-19 pandemic.

Several systematic reviews have summarized studies that evaluate HCoV epidemiology; all were conducted in the early months of the COVID-19 pandemic and hence did not evaluate its impact on HCoV epidemiology [13, 75, 76]. Park et al [13] and Li et al [75] focused on characterizing the seasonality of HCoVs, and only included studies that reported proportions of HCoV infection per month or season (22 and 40 studies, respectively). Li et al [75] focused on community studies and thus excluded those that recruited patients from secondary or tertiary care. Both reviews found that peak HCoV activity predominantly occurred in winter months at temperate sites, though year-round activity was observed in China [75]. Additionally, Li et al identified significant cocirculation of HCoVs during influenza virus and respiratory syncytial virus seasons at temperate sites, with less overlap observed in China and tropical sites. A third systematic review and meta-analysis that included 128 studies derived a pooled prevalence of HCoV infection of 5.21% (95% confidence interval, 4.62–5.83), but the search strategy and clinical syndromes eligible for study inclusion were not stated [76]. Importantly, none of the existing systematic reviews reported proportion test positivity or seasonality for individual HCoV species, reporting only aggregated data for all HCoVs. Furthermore, none included case-control studies to ascertain the clinical significance of a positive HCoV test. In contrast, we assessed test positivity for individual HCoV species, where possible, from studies conducted in both community and health care settings. Just over one third of included studies reported results of individual HCoV species, which found OC43 to be the most prevalent and that the prevalence of individual HCoVs varied by age group (pediatric vs adult). To avoid including biased estimates on the contribution of HCoVs to mild and severe ARI, we restricted studies to those that included a minimum of 12 consecutive months and estimated median prevalence for studies that covered multiples of 12-month periods only. Seasonality was only investigated in studies that included at least 100 HCoV-positive samples annually because ascertaining seasonal patterns with fewer samples is likely to be challenging. In agreement with Park et al [13] and Li et al [75], we found that winter months were associated with the highest HCoV test positivity. Nevertheless, the few studies from nontemperate settings suggest that HCoV seasonality may differ in these regions [45, 47]. Moreover, distinct seasonality and periodicity were observed in studies that evaluated individual HCoV species, thus highlighting the importance of evaluating individual species separately.

A detailed understanding of the epidemiology and seasonality of HCoVs may help us predict how SARS-CoV-2 may circulate in coming years. Nevertheless, it is important to appreciate the key differences between SARS-CoV-2 and HCoVs; first, children appear to be less susceptible [77, 78] to SARS-CoV-2 infection and have less severe disease than adults, whereas greater susceptibility and burden of infection in children, compared to adults, have been reported for HCoVs [20] and other respiratory viruses [79]. Indeed, it has been postulated that milder COVID-19 in children may partly be due to preexisting cross-reactive immunity from more recent and frequent HCoV infections than adults [80]. This remains under debate because several studies have demonstrated cross-reactivity of HCoV antibodies with SARS-CoV-2 spike and nucleocapsid proteins, but they are nonneutralizing [81, 82]. Conversely, other studies support the hypothesis that cross-reactive antibodies from prior exposure to HCoVs lead to neutralization and protection against SARS-CoV-2 [8]. Second, although a high proportion of the global population have received 1 or more vaccines against SARS-CoV-2, there are currently no vaccines against HCoVs. COVID-19 vaccination, however, has been shown to produce cross-reactive antibodies to the beta-coronaviruses, OC43 and HKU1, but not the alpha-coronaviruses [83].

The studies included in this review had several limitations. The epidemiology and seasonality of HCoVs should ideally be assessed through studies with sufficient sample sizes that recruit year-round and span multiple years. Although we only included studies with a minimum of 100 cases, the number of individual HCoVs reported was often low. Several studies found that different HCoV species had distinct peak activity [43, 50, 84]; thus, aggregation of HCoVs could have masked seasonal trends. We were unable to assess seasonality in the majority of included studies (>90%) because of low HCoV case numbers or lack of seasonal information. Studies that evaluated seasonality did not include assessment of extrinsic drivers, such as climactic conditions (eg, temperature, humidity) and associated behavioral changes (eg, increased time spent indoors during winter and rainy season). Few studies included contemporaneous asymptomatic controls, which would clarify the role of HCoVs in clinical disease. Furthermore, the studies employed varying definitions of respiratory illness, differing age categories, as well as time periods, that precluded the conduction of a meta-analysis. The underrepresentation of studies from the Southern Hemisphere, particularly from tropical and subtropical regions, means that the seasonal patterns of HCoVs in these regions remain unclear. Moreover, around 1 in 6 studies had a medium or high risk of bias; unclear reporting of participant demographics, clinical information, and the lack of clear inclusion criteria were the key sources of bias. Because of the substantial number of manuscripts on SARS-CoV-2, we pragmatically elected to exclude “SARS-CoV-2”' and “COVID-19”' in our literature search, and therefore could have omitted relevant studies during the pandemic. Last, studies may have been omitted as preprints, gray literature, and articles written in languages other than English were not included.

## CONCLUSION

This review summarized existing studies on the epidemiology and seasonality of individual HCoV species. OC43 has the highest prevalence for all ages, though test positivity of individual HCoVs varied by age. Seasonality and periodicity also differed by HCoV species. HCoVs typically show winter seasonality in temperate regions, but burden and seasonality of HCoV remain unclear in Africa, Central/Latin America, and South Asia. Several studies have demonstrated perturbations in the seasonality of HCoVs, as well as other viruses following the A(H1N1)2009 and COVID-19 pandemic. Ongoing surveillance is key to characterize the circulation of seasonal respiratory viruses, including HCoVs, as we transition out of the COVID-19 pandemic. The inclusion of healthy contemporaneous controls will be important to better understand the contribution of HCoVs to clinical illness.

## Supplementary Material

ofae418_Supplementary_Data

## Supplementary Data

Supplementary materials are available at *Open Forum Infectious Diseases* online. Consisting of data provided by the authors to benefit the reader, the posted materials are not copyedited and are the sole responsibility of the authors, so questions or comments should be addressed to the corresponding author.

## Notes

***Author Contributions*. Rory Wilson:** Investigation, Data curation, Writing—Original draft. **Dory Kovacs:** Investigation, Formal Analysis, Data curation, Writing—Original draft, Visualization. **Mairi Crosby:** Methodology, Investigation, Writing—Original draft. **Antonia Ho:** Conceptualization, Investigation, Writing—Original draft, Supervision.

***Acknowledgments*.** We would like to acknowledge Scott Ramsay and Paul Cannon at the University of Glasgow Library for their assistance with the literature search.

***Data availability statement***. All data are from published papers.

***Patient consent for publication statement*.** This review did not require approval from an ethical committee or individual patient consent. This is due to the data being completely anonymous and already available in the public domain.

***Provenance and peer review*.** Not commissioned; externally peer reviewed.

***Financial support*.** The authors have not declared a specific grant for this research from any funding agency in the public, commercial, or not-for-profit sectors.
