# Supplementary material for: Global Epidemiology and Seasonality of Human Seasonal Coronaviruses: A Systematic Review
Source: Open Forum Infect Dis. 2024 Jul 18;11(8):ofae418. doi: 10.1093/ofid/ofae418 (PMC11304597; doi:10.1093/ofid/ofae418)
Supplement: ofae418_Supplementary_Data [file ofae418_supplementary_data.docx]

**Supplementary Material & Appendices**

[Supplementary Tables 2](#_Toc169479112)

[**Supplementary Table 1.** Characteristics of included studies 2](#_Toc169479113)

[**Supplementary Table 2**: Studies that included control subjects 12](#_Toc169479114)

[**Supplementary Table 3.** Prevalence of human coronaviruses and coinfections reported in included studies 15](#_Toc169479115)

[**Supplementary Table 4.** Prevalence of human coronaviruses reported in included studies 20](#_Toc169479116)

[Appendix 21](#_Toc169479117)

[**Appendix 1:** Search Terms 21](#_Toc169479118)

[**Appendix 2.** Risk of Bias 22](#_Toc169479119)

[**Case series** 22](#_Toc169479120)

[**Cohort studies** 29](#_Toc169479121)

[**Case-control studies** 30](#_Toc169479122)

# **Supplementary Tables**

## **Supplementary Table 1.** Characteristics of included studies

| Author (year) | Country (or countries) | Study setting* | Number of Site | Controls (Y/N) | Study design | Clinical criteria for testing (original wording) | Age groups | Sex (% male or m:f ratio) | Study years | Complete 12 month period (Y/N) | Sample size  (n participants) | OC43 | NL63 | 229E | HKU1 |
| --- | --- | --- | --- | --- | --- | --- | --- | --- | --- | --- | --- | --- | --- | --- | --- |
| Agustiningsih (2012) | Indonesia | Secondary | Multi | N | Case series (retrospective) | ARI | All | 49.1% | 2008–2009 | Y | 230 | N | Y (grouped with 229E) | Y (grouped with NL63) | N |
| Ahn (2014) | South Korea | Secondary | Single | N | Case series (prospective) | ARI, ARDS, pneumonia | Paediatric | 60% | 2010–2011 | Y | 1,528 | Y | N | Y | N |
| Akagi (2021) | Japan | Secondary | Single | N | Case series (prospective) | ARI | Adult | 47% | 2014–2019 | Y | 121 | Y | Y | Y | Y |
| Al-Khannaq (2016) | Malaysia | Primary | Single | N | Case series (prospective) | URTI | Adult | 51.5% (reported for HCoV positive only) | 2012–2013 | Y | 2,060 | N | Y | Y | N |
| Al-Romaihi (2020) | Qatar | Primary, Secondary | Multi | N | Case series (prospective) | ILI | Paediatric | 42.9% male, 33.3% female, 23.8% missing | 2012–2017 | Y | 33,404 specimens | Y | Y | Y | Y |
| Ali (2016) | Pakistan | Community | Multi | N | Case series (prospective) | Severe Pneumonia | Paediatric | 50.1% | 2011-2014 | N | 817 | Y | Y | Y | Y |
| Altay-Kocak (2022) | Turkey | Tertiary | Single | N | Case series (prospective) | ARTI | Paediatric | 55% | 2016-2019 | N | 1,592 | Y | Y | Y | Y |
| Amer (2016) | Saudi Arabia | Tertiary | Single | N | Case series (retrospective) | RTI | Paediatric | 60% | 2008-2009 | N | 174 | Y (grouped with HKU1) | Y (grouped with 229E) | Y (grouped with NL63) | Y (grouped with OC43) |
| Anand (2020) | India | Secondary | Multi | N | Case series (prospective) | ARI | All | 57.5% | 2017–2019 | Y | 513 | Y | Y | Y | Y |
| Angeles Marcos (2006) | Spain | Secondary | Single | N | Case series (prospective) | CAP | Adult | 58% | 2003-2004 | N | 198 | Y | N | Y | N |
| Annan (2016) | Germany, Ghana | "Ghana: Community Germany: Secondary" | Multi | N | Case series (retrospective) | ARI | Paediatric |  | "Ghana: 2008–2009 Germany: 2007–2008" | Y | 1,713 | Y | Y | Y | Y |
| Arden (2005) | Australia | Secondary | Multi | N | Case series (retrospective) | ARI | All | NR | 2001-2004 | N | 840 | Y | N | Y | N |
| Aygun (2020) | Turkey | Secondary | Single | N | Case series (retrospective) | LRTI | Paediatric | 56.6% | 2012-2016 | N | 422 | Y | Y | Y | N |
| Azziz-Baumgartner (2021) | Ecuador | Secondary | Single | N | Birth cohort | ARI | Paediatric | 51% | 2011–2014 | N | 2,376 | Y | Y | Y | Y |
| Baillie (2021) | South Africa, Zambia | Secondary | Multi | Y | Case-control | Pneumonia | Paediatric | NR | 2011–2014 | N | 3,187 | Y | Y | Y | Y |
| Beka (2013) | Turkey | Secondary | NR | N | Case series (prospective) | ARTI | Paediatric | 57% | 2006-2007 | N | 109 | Y | Y | Y | Y |
| Bellei (2008) | Brazil | Primary and Secondary | Multi | N | Case series (prospective) | ARI and ILI | Adult | 36% | 2001-2003 | N | 420 | Y | N | Y | NR |
| Berce (2015) | Slovenia | Secondary | Single | N | Case series (retrospective) | LRTI | Paediatric | 52% | 2012-2013 | N | 278 | Y | Y | Y | Y |
| Berkley (2010) | Kenya | Secondary | Single | Y | Case series (prospective) & case-control | URTI & pneumonia | Paediatric | 59% | 2007 | Y | 759 | Y | Y | Y | Y |
| Bimouhen (2022) | Morocco | Primary and Secondary | Multi | N | Case series (prospective) | SARI and ILI | All | 48.70% | 2014–2016 | N | 2,009 | Y | Y | Y | Y |
| Bouvier (2018) | USA | Primary, Secondary | Multi | N | Case series (prospective) | ILI | All | 49% (reported for HCoV positive only) | 2009–2014 | NR | 902 | Y | Y | Y | Y |
| Brini (2017) | Tunisia | Secondary | Single | N | Case series (prospective) | ARTI | Paediatric | 64% | 2013–2014 | N | 372 | Y | Y | Y | Y |
| Brini (2019) | Tunisia | Secondary | Single | N | Case series (retrospective) | ARI | Paediatric | 62.9% | 2013–2014 | N | 515 | Y | Y | Y | Y |
| Brittain-Long (2011) | Sweden | Primary, Secondary | Multi | N | Case series (retrospective) | NR | All | 48.7% | 2006–2009 | Y | 7,220 | Y | Y | Y | N |
| Cabeca (2013) | Brazil | Community, Primary, Secondary | Single | Y (part of study) | Case series (prospective) | ILI, ARI, hospitalised patients suspected of A/H1N1pdm09 infection, LRTI | All | NR | 2001–2010 | N | 1,137 | Y | Y | Y | Y |
| Calvo (2020) | Spain | Secondary | Single | N | Case series (prospective) | ARI | Paediatric | 52% (reported for HCoV positive only) | 2005–2018 | NR | 5,131 | Y | Y | Y | Y |
| Canducci (2008) | Brazil | Secondary | Single | N | Case series (prospective) | ARI | Paediatric | 55% | 2004–2006 | Y | 322 | Y | Y | Y | Y |
| Cebey-López (2015) | Spain, UK | Secondary | Multi | N | Prospective cohort | ALRTI | Paediatric | Spain: 1.7, UK: 0.94 | Spain: 2011-2013, UK: 2009-2012 | Spain: Y, UK: NR | Spain: 204, UK: 97 | Y | Y | Y | N |
| Chen (2022) | China | Secondary | Multi | N | Case series (retrospective) | Pneumonia | Paediatric | 2.09:1 (reported for HCoV positive only) | 2018 | NR | 1,358 | Y | Y | Y | Y |
| Chen Y (2021) | Singapore | Primary | Multi | N | Case series (prospective) | URTI | Adult | 65% | 2007-2013 | N | 2,057 | Y | Y | Y | Y |
| Chiu (2005) | China (Hong Kong) | Secondary | Single | N | Case series (prospective) | ARI | Paediatric | 60% (reported for HCoV positive only) | 2001-2002 | Y | 587 | Y | Y | Y | N |
| Choi (2021) | South Korea | Secondary | Single | N | Case series (retrospective) | ARI | Paediatric | 58% | 2015–2019 | Y | 9,589 | Y | Y | Y | N |
| Chonmaitree (2008) | USA | Primary and community | Multi | N | Case series (prospective) | URI | Paediatric | 51% | 2003-2006 | N | 864 | Y | N | Y | N |
| Chow (2022) | USA | Community | Multi | N | Prospective cohort | ARI | Adult | 60% | 2019-2021 | Y | 14,464 | Y | Y | Y | Adult |
| Ciotti (2020) | Italy | Tertiary | Single | N | Case series (retrospective) | RTI | Adult | 60.70% | 2016-2019 | N | 539 | Y | Y | Y | Y |
| Cui (2015) | China | Primary | Single | N | Case series (prospective) | ARI | Paediatric | 62.3% | 2010–2011 | Y | 1,074 | N | N | N | N |
| Dare (2007) | Thailand | Secondary | Multi | Y | Case-control | pneumonia & ILI | All | NR | 2003–2005 | Y | 7,449 | Y | Y | Y | Y |
| Das (2015) | France | Secondary | Multi | N | Case series (prospective) | CAP | Adult | 49% | 2011-2012 | Y | 254 | Y | Y | Y | N |
| De Conto (2019) | Italy | Tertiary | Single | N | Case series (prospective) | ARI | Paediatric | 55% (of those virus positive) | 2012–2015 | Y | 2,575 | Y | Y | Y | Y |
| Delangue (2014) | Bolivia | Secondary | Multi | N | Case series (retrospective) | ILI | All | 45.6% | 2010–2012 | N | 564 | Y (grouped with 229E) | N | Y (grouped with OC43) | N |
| Diederen (2009) | Netherlands | Secondary | Single | N | Case series (prospective) | Pneumonia | Adult | NR | 1998–2000 | Y | 242 | Y (grouped with 229E) | N | Y (grouped with OC43) | N |
| Do (2011) | Vietnam | Tertiary | Single | N | Case series (prospective) | ARI | Paediatric | 56% | 2004-2008 | N | 309 | Y | N | Y | Y |
| Dos Santos Ferreira (2019) | Brazil | Secondary | Multi | N | Case series (prospective) | CAP | Paediatric | 57% | 2014-2016 | N | 150 | Y | Y | N | N |
| Dyrdak (2021) | Sweden | Secondary | Single | N | Case series (retrospective) | NR (routine diagnostics) | All | NR | 2009–2020 | N | 55,190 | Y | Y | Y | Y |
| Essa (2015) | Kuwait | Secondary | Single | N | Case series (prospective) | URTI & LRTI | All | 57.2% (of those virus positive) | 2010–2013 | N | 735 | Y | Y | Y | N |
| Etenna (2014) | Gabon | Primary and secondary | Multi | N | Case series (prospective) | ILI | All | 49.50% | 2010–2011 | N | 1,041 | Y | Y | Y | Y |
| Fagbo (2015) | Saudi Arabia | Tertiary | Single | N | Case series (prospective) | ARI | Paediatric | m:f = 3:2 | 2012–2013 | Y | 2,235 | Y | Y | Y | N |
| Fairchok (2010) | USA | Community | Multi | N | Prospective cohort | >=2 symptoms from: cough, rhinorrhoea, wheezing, fever, and nasal congestion | Paediatric | 53% | 2006–2008 | N | 119 | Y | Y | Y | Y |
| Faye (2023) | Senegal | Primary, Secondary | Multi | N | Case series (retrospective) | ILI, SARI | All | 49.7% | 2012-2020 | N | 9337 | Y | Y | Y | Y |
| Feng (2014) | China | Secondary | Multi | N | Surveillance | ALRI | All | 64.2% | 2009–2013 | N | 28,369 | NR | NR | NR | NR |
| Ferreira (2019) | Brazil | Secondary | Multi | N | Case series (prospective) | Pneumonia | Paediatric | 56.7% | 2014–2016 | N | 150 | Y | Y | N | N |
| Fillatre (2018) | France | Secondary | Single | N | Case series (retrospective) | ARI | Paediatric | NR | 2012–2016 | Y | 3,199 | Y | Y | Y | Y |
| Frutos (2022) | Nicaragua | Primary | Multi | N | Prospective cohort | ARI, ALRI | Paediatric | 49.3% | 2011–2016 | Y | 2,576 | Y | Y | Y | Y |
| Fu (2015) | China | Secondary | Single | N | Surveillance | ILI | All | 51.8% | 2011–2013 | Y | 1,970 | Y | Y | Y | Y |
| Furuse (2010) | Philippines | Secondary | Multi | N | Surveillance | ILI | All | 49.1% | 2006–2007 | Y | 411 | Y | NR | NR | Y |
| Gagneur (2008) | France | Secondary | Single | N | Surveillance | NR | Paediatric | 65% | 1998 | Y | 244 | N | N | Y | N |
| Garbino (2006) | Switzerland | Secondary | Multi | N | Case series (prospective) | ARI | All | 63% | NR | N | 540 | Y | Y | Y | Y |
| Garbino (2009) | Switzerland | Secondary | Multi | N | Prospective cohort | RTI | Adult | 61% | 2003-2006 | N | 229 | Y | Y | Y | Y |
| Gaunt (2010) | UK | Secondary | Single | N | Case series (retrospective) | NR | All | NR | 2006–2009 | Y | 7,383 | Y | Y | Y | Y |
| Gil (2018) | Portugal | Tertiary | Single | N | Case series (prospective) | LRTI | Paediatric | 56.1% | 2012-2015 | N | 451 | Y | Y | Y | N |
| Goes (2019) | Brazil | Community | Single | N | Case series (prospective) | ARI | Paediatric | 48.2% | 2005–2006 | Y | 282 | Y | Y | Y | Y |
| Goktas (2016) | Turkey | Secondary | Multi | N | Case series (retrospective) | ARI | All | 52.5% | 2014–2015 | Y | 845 | Y | Y | Y | Y |
| Graat (2003) | Netherlands | Community | Multi | Y | Case series (prospective) | ARI | Elderly | 45% | 1998-1991 | Y | 97 | Y | Y | N | N |
| Guerrier (2013) | Cambodia | Secondary | Multi | N | Case series (prospective) | ALRTI | Paediatric | 58% | 2007-2010 | N | 1,006 | Y | Y | Y | Y |
| Haddadin (2021) | Jordan | Secondary | Single | N | Case series (prospective) | ARI, pneumonia, ARDS | Paediatric | 59% (reported for HCoV positive only) | 2010–2013 | Y | 3,168 | Y | Y | Y | Y |
| Hajjar (2010) | Saudi Arabia | Secondary | Single | N | Case series (prospective) | ARI | Paediatric | NR | 2007–2008 | N | 489 | N | Y | N | N |
| Han (2007) | South Korea | Secondary | Single | N | Case series (prospective) | ARI | Paediatric | m:f = 1.8 (only reported for NL63 positive) | 2004–2006 | Y | 827 | N | Y | N | N |
| Hara (2015) | Japan | Primary | Single | N | Case series (retrospective) | ARI | Paediatric | Cases: 33% | 2008-2011 | N | 495 | Y | Y | Y | Y |
| Hasuwa (2020) | Japan | Secondary | Multi | N | Case series (prospective) | ALRI | Paediatric | 52% | 2013–2015 | Y | 373 | Y | N | Y | N |
| Hatem (2019) | Egypt | Tertiary | Single | N | Case series (prospective) | SARI | Adult | 47% | 2010–2014 | N | 3,207 | NR | NR | NR | NR |
| Hautala (2020) | Finland | Secondary | Single | N | Case series (prospective) | ARI | Paediatric | 57% | 2013–2017 | N | 1,899 | Y | Y | Y | N |
| Hawkes (2021) | Canada | Secondary | Multi | N | Surveillance | NR (routine diagnostics) | Paediatric | 51.1% | 2005–2017 | N | 37,719 | Y | Y | Y | Y |
| Heimdal (2019) | Norway | Secondary | Single | Y | Surveillance | ARI | Paediatric | 60.7% | 2006–2015 | N | 3,831 | Y | Y | Y | Y |
| Heimdal (2022) | Norway | Secondary | Single | N | Case series (prospective) | RTI | Paediatric | m:f = 1.3:1 (HCOV only) | 2006-2017 | Y | 4,312 | Y | Y | Y | N |
| Helou (2022) | Lebanon | Secondary | Multi | N | Case series (prospective) | CARTI | All | NR | 2017-2018 | N | 100 | Y | Y | Y | Y |
| Hoffmann (2012) | Madagascar | Secondary | Single | N | Case series (prospective) | ARI | Paediatric | NR | 2010–2011 | Y | 295 | Y | Y | Y | Y |
| Hu (2014) | China | Secondary | Multi | N | Case series (prospective) | ARI | Adult | 47.8% | 2011–2012 | Y | 559 | Y | N | N | N |
| Huang (2013) | China | Secondary | Single | N | Case series (prospective) | ARI | Paediatric | 66% | 2011-2011 | N | 279 | Y | Y | Y | Y |
| Huang, S-H (2017) | Taiwan | Secondary | Single | N | Case series (retrospective) | Pneumonia & ILI | All | NR | 2010–2011 | N | 282 | N | Y | N | N |
| Huang, X-B (2020) | China | Secondary | Multi | N | Case series (prospective) | ARI | All | m:f = 1.58 | 2009–2018 | Y | 22,680 | Y | Y | Y | Y |
| Ieven (2018) | Europe | Primary | Multi | Y | Case-control | LRTI, CAP | Adult | 40% | 2007-2010 | N | 3,104 | Y | Y | Y | N |
| Jain (2015) | USA | Secondary | Multi | Y | Surveillance | Pneumonia | Adult | 49% | 2010–2012 | N | 2,320 | Y | Y | Y | Y |
| Jain (2015) | USA | Secondary | Single | Y | Surveillance | Pneumonia | Paediatric | 55% | 2010–2012 | N | Cases: 2358 Controls: 521 | Y | Y | Y | Y |
| Jean (2013) | Canada | Tertiary | Single | Y | Case-control | ARI & pneumonia | Paediatric | NR | 2009–2010 | Y | 3,847 | Y | N | Y | N |
| Jeon (2019) | UAE | Secondary | Single | N | Case series (prospective) | ARI | All | 53.8% | 2015-2018 | N | 1,362 | Y | Y | Y | N |
| Jevsnik (2012) | Slovenia | Secondary | Single | N | Case series (retrospective) | ARI | Paediatric | 57% | 2007–2008 | Y | 592 | Y | Y | Y | Y |
| Jin (2010) | China | Secondary | Single | N | Case series (prospective) | ARI | Paediatric | 53% | 2006–2008 | Y | 645 | N | N | N | Y |
| Jin (2012) | China | Secondary | Single | N | Case series (prospective) | ALRTI | Paediatric | m:f = 1.9 | 2006–2009 | Y | 813 | N | Y | N | Y |
| Jo (2022) | South Korea | Secondary | Multi | N | Case series (retrospective) | NR | Paediatric | 60.6% | 2015-2019 | Y | 1,096 | Y | Y | Y | Y |
| Johnstone (2008) | Canada | Secondary | Multi | N | Case series (prospective) | Pneumonia | Adult | 51% | 2004–2006 | Y | 193 | Y | Y | Y | N |
| Kadjo (2018) | Cote d'Ivore | Secondary | Multi | N | Case series (prospective) | ILI & SARI | Paediatric | 54% | 2013 | Y | 1,059 | Y | N | Y | N |
| Kenmoe (2016) | Cameroon | Secondary | Single | N | Case series (prospective) | SARI | Paediatric | 51.9% | 2011–2013 | Y | 347 | Y | Y | Y | Y |
| Khalifa (2018) | Tunisia | Secondary | Single | N | Case series (retrospective) | ARI | Paediatric | 62.9% | 2013–2014 | N | 515 | Y | Y | Y | Y |
| Khamis (2012) | Oman | Secondary | Single | N | Case series (prospective) | ILI | Paediatric | 59% | 2007–2008 | Y | 259 | Y | Y | Y | Y |
| Khomenko (2021) | Ukraine | Secondary | Multi | N | Case series (prospective) | URTI & LRTI | Paediatric | 57.8% | 2018–2020 | N | 487 | Y (grouped with HKU1) | Y (grouped with 229E) | Y (grouped with NL63) | Y (grouped with OC43) |
| Killerby (2018) | USA | Clinics and laboratories (passive surveillance) | Multi | N | Surveillance | NR (routine diagnostics) | All | 50.6% (reported for HCoV positive only) | 2014–2017 | Y | 854,575 | Y | Y | Y | Y |
| Kim, H-C (2016) | South Korea | Tertiary | Single | N | Case series (retrospective) | Pneumonia | Adult | 86.40% | 2010–2012 | Y | 477 | Y | Y (grouped with 229E) | Y (grouped with NL63) | N |
| Kim, J-M (2018) | South Korea | Secondary | Multi | N | Surveillance | ARI | All | NR | 2013–2015 | Y | 36,915 | Y | Y | Y | N |
| Kim, JM (2020) | South Korea | Secondary | Single | N | Case series (retrospective) | NR | Paediatric | NR | 2012–2018 | Y | 9,010 | Y | N | Y | N |
| Kim T (2021) | South Korea | Tertiary | Multi | N | Case series (retrospective) | ARI | All | 58% | 2018-2020 | N | 24,311 | Y | Y | Y | N |
| Kiyuka (2018) | Kenya | Community, Secondary | Multi | N | Surveillance | Pneumonia | Paediatric | NR | 2008–2014 | N | 5,573 | N | Y | N | N |
| Klivleyeva (2023) | Kazakhstan | Primary, Secondary | Multi | N | Case series (retrospective) | ARI (SARS-CoV-2 negative) | All | NR | 2018–2022 | NR | 4,712 | Y | Y | Y | Y |
| Koetz (2006) | Sweden | Primary, Secondary | Multi | N | Surveillance | ARI | Paediatric | 52% | 2003–2005 | N | 221 | N | Y | N | N |
| Koker (2019) | Turkey | Secondary | Single | N | Case series (prospective) | ARI | Paediatric | 53.7% | 2014–2017 | N | 108 | Y | Y | Y | Y |
| Komabayashi (2021) | Japan | Secondary | Single | N | Case series (retrospective) | NR | Paediatric | 52% | 2010–2017 | Y | 7,538 | Y | N | N | N |
| Kong (2021) | China | Secondary | Multi | N | Case series (retrospective) | ILI, SARI | All | NR | 2015-2020 | N | 4541 | Y (Grouped HKU1) | Y | Y (Grouped with NL63) | All |
| Kozak (2020) | Canada | Secondary | Single | N | Case series (retrospective) | ARI | Adult | 44.7% | 2010–2016 | Y | 5,308 | Y | Y | Y | Y |
| Kumar (2017) | India | Secondary | Single | N | Birth cohort | ARI | Paediatric | 54.5% | 2012-2014 | N | 310 | Y | Y | Y | Y |
| Kumar (2020) | India | Secondary | Single | N | Birth cohort | ARI | Paediatric | 54.5% | 2012–2014 | NR | 310 | Y | Y | Y | Y |
| Kuribayashi (2022) | Japan | Secondary | Single | N | Case series (retrospective) | NR | Paediatric | 55% | 2021 | Y | 1,769 | Y | Y | Y | Y |
| Kurskaya (2018) | Russia | Secondary | Single | N | Case series (prospective) | ARI | Paediatric | 52.8% | 2013-2017 | N | 1,560 | Y | Y | Y | Y |
| Kuypers (2007) | USA | Secondary | Single | N | Case series (prospective) | ARI | Paediatric | NR | 2003–2004 | Y | 1,043 | Y | Y | Y | Y |
| Lambert (2007) | Australia | Community | Multi | N | Case series (prospective) | ARI | Paediatric | 49% | 2003-2004 | N | 730 | N | Y | N | N |
| Lau (2006) | China (Hong Kong) | Secondary | Multi | N | Case series (prospective) | ARI | All | NR | 2004–2005 | Y | 4,181 | Y | Y | Y | Y |
| Le (2020) | Vietnam | Secondary | Multi | N | Case series (prospective) | SARI | Adult | 36.8% male, 0.8% unknown | 2017–2019 | Y | 348 | N | N | Y | Y |
| Lee (2013) | South Korea | Community | Multi | N | Case series (prospective) | ARI | Adult | NR | 2007-2008 | N | 1,985 | N | N | N | Y |
| Leli (2021) | Italy | Secondary | Single | N | Case series (retrospective) | ARI | Adult | 58.9% | 2016–2020 | N | 572 | Y | Y | Y | Y |
| Li (2018) | China | Secondary | Multi | N | Case series (prospective) | ARI | All | 66.3% | 2011-2015 | N | 2,768 | NR | NR | NR | NR |
| Li (2021) | China | Secondary | Single | N | Case series (prospective) | SARI | Adult | 48.9% | 2017–2018 | Y | 387 | Y | Y | Y | Y |
| Li Y-T J (2019) | China | Tertiary | Single | N | Case series (prospective) | SALRTI | Paediatric | 63% | 2015-2018 | N | 659 | Y | Y | Y | Y |
| Litwin (2014) | USA | Secondary, Tertiary | Single | N | Case series (retrospective) | ARI | All | m:f = 1.42 | 2011–2012 | Y | 939 | Y | Y | Y | Y |
| Liu, P (2017) | China | Community | Multi | N | Case series (prospective) | ARI | Paediatric | 68% | 2014–2015 | N | 3,298 | Y | Y | Y | Y |
| Liu, T (2017) | China | Secondary | Multi | N | Case series (prospective) | ARI | All | 57.3% | 2011–2013 | Y | 607 | Y (grouped with HKU1) | Y (grouped with 229E) | Y (grouped with NL63) | Y (grouped with OC43) |
| Liu, WK (2014) | China | Secondary | Multi | N | Case series (prospective) | ARI | Paediatric | m:f = 1.92 | 2009–2012 | Y | 4,242 | Y | Y | Y | Y |
| Low (2022) | Malaysia | Community, Secondary | Multi | N | Case series (retrospective) | URTI, LRTI | Paediatric | 51.9% | 2015–2019 | Y | 23,306 | Y | Y | Y | Y |
| Lu (2013) | China | Secondary | Single | N | Case series (prospective) | ARI | Adult | 49% | 2009–2010 | Y | 596 | Y | N | Y | N |
| Malhotra (2016) | India | Secondary | Single | N | Case series (prospective) | SARI | Paediatric | NR | 2012-2013 | Y | 155 | Y | Y | Y | Y |
| Matienzo (2020) | USA | Secondary | Multi | N | Case series (prospective) | ILI | All | NR | 2016–2018 | N | 1,315 | Y | Y | Y | Y |
| Matoba (2015) | Japan | Secondary | Multi | N | Case series (retrospective) | ARI | Paediatric | NR | 2010–2013 | Y | 4,342 | Y | Y | Y | Y |
| Matsuno (2019) | Brazil | Tertiary | Single | N | Case series (prospective) | ALRI | Paediatric | 38% | 2008-2009 | N | 279 | Y | Y | Y | Y |
| Mohammadi (2020) | Iran | Secondary | Single | N | Case series (prospective) | ARI | Paediatric | 55.8% | 2018–2019 | N | 138 | N | Y | N | N |
| Monto (2020) | USA | Community | Multi | N | Prospective cohort | ARI | All | NR | 2010–2018 | Y (from 2014-2018) | 7,469 | Y | Y | Y | Y |
| Nascimento-Carvalho (2018) | Brazil | Secondary | SIngle | N | Prospective cohort | Non-severe CAP | Paediatric | 53.1% | 2006–2011 | N | 774 | Y | Y | Y | N |
| Nguyen (2016) | Vietnam | Community | Multi | N | Case series (prospective) | ILI | All | 44% | 2008-2013 | N | 435 | Y | Y | Y | Y |
| Nickbakhsh (2016) | UK | Primary, Secondary | Multi | N | Case series (retrospective) | NR (routine diagnostics) | All | 51% | 2005–2013 | Y | 44,230 | Y | Y | Y | Y |
| Nickbakhsh (2020) | UK | Primary, Secondary, Tertiary | Single | N | Case series (retrospective) | NR (routine diagnostics) | All | 48.4% | 2005–2017 | N | 64,948 | Y | Y | Y | N |
| Nicol (2021) | South Africa | Community | Multi | Y | Case-control | Pneumonia | Paediatric | 55% | 2012–2015 | Y | 885 | Y | Y | Y | Y |
| Noyola (2019) | Mexico | Secondary, Tertiary | Multi | N | Prospective cohort | ILI | All | 42.6% | 2010-2014 | N | 3,926 | Y | Y | Y | Y |
| Nunes (2014) | South Africa | Secondary | Single | N | Case series (retrospective) | LRTI | Paediatric | 57.3% | 2000–2002 | Y | 1,460 | Y | Y | Y | Y |
| Nyiro (2018) | Kenya | Primary | Multi | N | Case series (prospective) | ARI | All | 42.3% | 2016–2016 | N | 5,647 | Y | Y | Y | Y |
| Ortiz-Hearnandez (2019) | Mexico | Primary and Secondary | Multi | N | Case series (prospective) | ILI | Paediatric | 55.8% | 2010-2014 | N | 1,486 | Y | Y | Y | Y |
| Otieno (2020) | Kenya | Secondary | Single | N | Prospective cohort | Pneumonia | Paediatric | 60.5% (reported for HCoV positive only) | 2007–2019 | Y | 7,957 | Y | Y | Y | N |
| Ottogalli (2020) | Argentina | Secondary | Multi | N | Case series (retrospective) | ARI | All | NR | 2011–2012 | Y | 631 | Y | N | Y | N |
| Owusu (2014) | Ghana | Community (rural) | Multi | Y | Case-control | URTI | All | cases: 43.7% controls: 41.8% | 2011–2012 | Y | 1,213 | Y | Y | Y | Y |
| Price (2019) | Australia | Community, Secondary | Multi | N | Surveillance | NR | All | male 51.2%, missing 5.1% | 2002–2017 | N | 58,114 | NR | NR | NR | NR |
| Qin (2021) | China | Secondary | Multi | Y | Case series (prospective) | CAP | All | 59.8% | 2016-2019 | N | 1,674 | Y | Y | Y | Y |
| Qu (2015) | China | Secondary | Multi | N | Case series (prospective) | Pneumonia | Adult | 56.6% | 2010–2012 | N | 954 | Y | Y | Y | Y |
| Radin (2014) | USA | Community (military) | Multi | N | Surveillance | ARI & SARI | All | 63% | 2011–2013 | N | 1,444 | Y | Y | Y | N |
| Razuri (2015) | Peru | Community | Multi | N | Prospective cohort | ILI | All | NR | 2010 | Y | 7,000 | Y | Y | Y | Y |
| Refay (2022) | Egypt | Tertiary | Multi | N | Case series (prospective) | ARI | Paediatric | 69% | 2017–2019 | N | 259 | Y | Y | Y | Y |
| Regamey (2008) | Switzerland | Community | Multi | N | Birth cohort | ARI | Paediatric | NR | 1999-2004 | N | 187 | Y | Y | Y | Y |
| Reina (2014) | Spain | Secondary | Single | N | Case series (prospective) | ARI | Adult | 54.1% (reported for HCoV positive only) | 2013–2014 | Y | 686 | Y | Y | Y | N |
| Ren (2009) | China | Primary | Single | N | Case series (prospective) | ARTI | Adult | 46% | 2005-2007 | N | 5,808 | Y | Y | Y | Y |
| Ren (2011) | China | Secondary | Single | N | Case series (prospective) | ARI | Adult | 46% | 2005–2009 | Y | 8,396 | Y | Y | Y | Y |
| Rhedin (2015) | Sweden | Secondary | Multi | Y | Case-control | CAP | Paediatric | NR | 2011-2014 | N | 361 | Y | Y | Y | Y |
| Sarna (2017) | Australia | Community | Multi | N | Birth cohort | ARI | Paediatric | 47.5% | 2010-2014 | Y | 8,100 | Y | Y | Y | Y |
| Sentilhes (2013) | Laos | Secondary | Multi | N | Case series (prospective) | ALRI | All | 49.7% | 2009–2010 | N | 292 | Y | Y | Y | Y |
| Seo (2014) | South Korea | Secondary | Single | N | Case series (retrospective) | ARI | All | NR | 2009-2012 | N | 3,865 | Y | N | Y | N |
| Shah (2022) | USA | Community | Multi | N | Surveillance | NR | All | 52.8% (reported for HCoV positive only) | 2014–2021 | N | 82,768 | Y | Y | Y | Y |
| Shan (2019) | China | Secondary | Single | N | Case series (prospective) | ARI | All | 59.60% | 2012–2015 | Y | 445 | Y | Y | Y | Y |
| Shi (2023) | China | Tertiary | Single | N | Case series (prospective) | ARI | Paediatric | 54% | 2021-2022 | N | 10,396 | Y | Y | Y | Y |
| Singleton (2010) | USA | Community, Secondary | Multi | Y | Case-control | LRTI | Paediatric | 51% (cases) | 2005–2007 | Y | 1073 (440 cases) | Y | Y | Y | Y |
| Sipulwa (2016) | Kenya | Secondary | Multi | N | Case series (retrospective) | ILI | All | 55% | 2009–2012 | Y | 417 samples | Y | Y | Y | Y |
| Sonawane (2019) | India | Tertiary | Single | N | Case series (prospective) | ALRTI | Paediatric | 65% | 2014-2015 | N | 100 | Y | Y | Y | Y |
| Sonmezer (2023) | Turkey | Secondary | Single | N | Case series (retrospective) | ARI | Adults | NR | 2015-2020 | Y | 4540 | Y | Y | Y | NT |
| Srinivasan (2013) | USA | Primary and Secondary | Multi | N | Case series (prospective) | URTI or LRTI | Paediatric | 55% | 2010-2011 | N | 253 | Y | Y | Y | Y |
| Sung (2009) | Hong Kong | Secondary | Single | N | Case series (prospective) | ARI | Paediatric | NR | 2005-2006 | Y | 475 | Y | N | Y | NR |
| Suzuki (2012) | Philippines | Secondary | Single | N | Case series (prospective) | Pneumonia | Paediatric | 54.5% | 2008–2009 | Y | 819 | Y | Y | N | N |
| Talbot (2009a) | USA | Secondary | Multi | N | Prospective cohort | URI and LRI | Paediatric | 55% | 2001–2003 | Y | 1,055 | Y | Y | Y | Y |
| Talbot (2009b) | USA | Primary | Single | N | Prospective cohort | URI and LRI | Paediatric | 51% | 1977–2001 | N | 1,830 | Y | Y | Y | N |
| Tao (2018) | China | Secondary | Single | N | Case series (prospective) | Pneumonia | Adult | 64.70% | 2015–2016 | N | 320 | NR | NR | NR | NR |
| Thaemboonlers (2005) | Thailand | Secondary | Single | N | Case series (prospective) | ALRI | Paediatric | 58% | 2002–2003 | N | 226 | Y | N | Y | N |
| Trombetta (2016) | Brazil | Tertiary | Single | N | Case series (retrospective) | SARI | Adult | 53% (reported for HCoV positive only) | 2012–2013 | Y | 755 | Y (grouped with HKU1) | Y (grouped with 229E) | Y (grouped with NL63) | Y (grouped with OC43) |
| Tsagarakis (2017) | Greece | Secondary | Single | N | Case series (retrospective) | NR | All | 55% | 2015–2016 | Y | 656 | Y | Y | Y | Y |
| Tuz (2022) | Turkey | Secondary | Single | N | Case series (retrospective) | URTI | Paediatric | 63% | 2015-2020 | Y | 2,606 | Y | Y | Y | N |
| Uddin (2018) | Nepal | Community | Multi | N | Prospective cohort | ARI | Paediatric | 55.4% (HCoV ARI cases) | 2011–2014 | Y | 3,505 | Y | Y | Y | Y |
| Vabret (2008) | France | Secondary | Single | N | Case series (prospective) | RTI | Paediatric | 63% | 2004-2005 | N | 1,002 | Y | Y | Y | Y |
| van der Zalm (2009) | Netherlands | Community | Single | N | Birth cohort | Cough, wheeze, with or without fever (>38°C) | Paediatric | 50.2% | 2003-2006 | Y | 305 | Y | Y | Y | N |
| Van Elden (2004) | Netherlands | Secondary | Single | Y | Case series (retrospective) | RTI | All | NR | NR | NR | 261 | Y | N | Y | N |
| van Gageldonk-Lafeber (2005) | Netherlands | Primary | Multi | Y | Case-control | ARTI and ILI | All | Cases: 47% Controls: 38% | 2000-2003 | Y | 1,082 | NR | NR | NR | NR |
| Varghese (2018) | USA | Community, Secondary | Multi | N | Case series (retrospective) | ARI | Paediatric | 51% (reported for HCoV positive only) | 2013–2014 | Y | 2582 | Y | Y | Y | Y |
| Venter (2011) | South Africa | Secondary | Multi | Y | Case series (retrospective) | ARI and healthy controls | Paediatric | 52.8% | 2006–2007 | Y | 1,637 | Y | Y | Y | Y |
| Visseaux (2017) | France | Secondary | Multi | N | Case series (retrospective) | NR | Adult | 60% | 2011–2016 | Y | 4,958 | N | Y | N | Y |
| Wansaula (2015) | USA | Secondary | Multi | N | Case series (prospective) | SARI | All | 52% | 2010–2014 | Y | 332 | Y | Y | Y | Y |
| Wertheim (2015) | Indonesia, Thailand, Vietnam | Secondary | Multi | N | Case series (retrospective) | ILI | All | 57% | 2008–2009 | Y | 1,222 | Y | Y | Y | Y |
| Wong-Chew (2017) | Mexico | Secondary | Multi | N | Case series (prospective) | CAP | Paediatric | 62% | 2010-2013 | N | 1,404 | Y | Y | Y | Y |
| Woo (2012) | China | Secondary | Single | N | Case series (retrospective) | ARI | All | NR | 2004–2005 | N | 6,272 | Y | Y | Y | Y |
| Xin (2012) | China | Secondary | Single | N | Case series (prospective) | ALRTI | Paediatric | 67.4% | 2006–2008 | Y | 878 | N | Y | N | N |
| Xu (2021) | China | Secondary | Multi | N | Case series (prospective) | ALRI | All | m:f = 1.79 | 2009–2013 | N | 28,369 | Y | Y | Y | N |
| Ye (2017) | China | Secondary | Multi | N | Case series (prospective) | ARI | Adult | 60.9% | 2012–2015 | Y | 967 | Y | Y | Y | Y |
| Ye (2023) | China | Secondary | Multi | N | Surveillance | ILI and SARI | All | Cases: 51.7% | 2016-2019 | Y | 15,677 | Y | Y | Y | Y |
| Yew (2019) | Malaysia | Secondary | Multi | N | Surveillance | ALRTI | Paediatric | 57.1% | 2014–2015 | Y | 397 | Y | Y | Y | Y |
| Yip (2016) | China (Hong Kong) | Secondary | Multi | N | Case series (retrospective) | ARI | All | m:f = 0.97 | 2008–2014 | Y | 8,275 | Y | Y | Y | Y |
| Yu (2012) | China | Secondary | Single | N | Case series (prospective) | ARTI | All | 43% | 2010-2011 | Y | 416 | Y | Y | Y | Y |
| Zeng (2018) | China | Secondary | Multi | N | Case series (retrospective) | URTI, LRTI, and ILI | Paediatric | m:f = 1.82 | 2009–2016 | Y | 11,399 | Y | Y | Y | Y |
| Zhang (2012) | China | Secondary | Single | N | Case series (prospective) | ARI | Paediatric | 68% | 2009-2010 | N | 164 | Y (grouped with HKU1) | Y (grouped with 229E) | Y (grouped with NL63) | Y (grouped with OC43) |
| Zhang, D (2014) | China | Secondary | Multi | N | Case series (prospective) | ARI | All | m:f = 1.48 | 2009–2012 | Y | 14,237 | NR | NR | NR | NR |
| Zhang, S-F (2018) | China | Secondary | Multi | N | Case series (prospective) | ARI | All | 61.10% | 2010–2015 | Y | 13,048 | Y | Y | Y | Y |
| Zhang, Y (2021) | China | Secondary | Single | N | Case series (retrospective) | ARI | Paediatric | 58.4% | 2017–2019 | N | 3,660 | Y | Y | Y | Y |
| Zhao (2019) | China | Secondary | Multi | N | Case series (prospective) | SARI | Paediatric | m:f = 1.5–1.8 (two hospitals) | 2008–2014 | N | 700 | Y | Y | Y | Y |
| Zhu (2021) | China | Secondary and Tertiary | Multi | N | Case series (prospective) | CAP | Paediatric | 60.7% | 2014-2016 | N | 2,721 | Y | Y | Y | Y |

**Abbreviations**: ARI, Acute Respiratory Infection; ALRTI, Acute Lower Respiratory Tract Infection; ARDS, Acute Respiratory Distress Syndrome; ARTI, Acute Respiratory Tract Infection; CAP, community-acquired pneumonia; HCoV, human coronaviruses; ILI, influenza-like illness; LRTI, lower respiratory tract infection; RTI, respiratory tract infections; URTI, upper respiratory tract infection; SARI, Severe Acute Respiratory Illness; Y, yes; N, no; NR, not reported.

*Primary care, Secondary care, Tertiary care or Community

## **Supplementary Table 2**: Studies that included control subjects

| **Author** | **Country** | **Sample size** | | **Age group** | **Case criteria** | **Controls** |  | **Likelihood of detecting seasonal HCoV** | | | | **Any difference in hCoV prevalence between cases & controls** |
| --- | --- | --- | --- | --- | --- | --- | --- | --- | --- | --- | --- | --- |
|  |  | **Cases** | **Controls** |  |  |  |  | **OC43** | **NL63** | **229E** | **HKU1** |  |
| **Baillie  (2021)** | South Africa & Zambia | 1537 | 1485 | <5 years | Hospitalised with WHO clinical criteria for severe or very-severe pneumonia | Age-frequency, HIV status and seasonally matched to cases. Symptomatic RTI (n=142) and asymptomatic group (n=1424) | Case | 36 (2.5) | 42 (2.9) | 8 (0.6) | 21 (1.5) | HCoV-HKU1 more frequently detected in asymptomatic controls compared to cases (3%, 40/1424 vs. 1%, 21/1447, p=0.017)  No difference between cases and RTI controls for any HCoV. |
|  |  |  |  |  |  |  | RTI | 4 (2.8) | 4 (2.8) | 0 (0) | 1 (0.7) |  |
|  |  |  |  |  |  |  | Asymptomatic | 49 (3.4) | 66 (4.6) | 5 (0.4) | 40 (2.8) |  |
| **Berkley  (2010)** | Kenya | 759 | 153 | <12 years | Hospitalised with WHO clinical criteria for severe or very-severe pneumonia | Mild URTI (n=96)  Asymptomatic children (n=57) | Case | 14 (1.8) | 10 (1.3) | 51 (6.7) | 1 (0.1) | Not reported. |
|  |  |  |  |  |  |  | Mild URTI | 2 (2.1) | 0 (0) | 3 (3.1) | 2 (2.1) |  |
|  |  |  |  |  |  |  | Asymptomatic | 4 (7) | 0 (0) | 1 (1.8) | 1 (1.8) |  |
| **Cabeca  (2013)** | Brazil | 1087 | 50 | All ages | ARI  General population (n=465)  Patients with comorbidities (n=410)  Hospitalised patients (n=212) | Asymptomatic adults | Case | 35 (3.2) | 64 (5.9) | 22 (2) | 7 (6.4) | Not reported. |
|  |  |  |  |  |  |  | Asymptomatic | 0 (0) | 0 (0) | 0 (0) | 0 (0) |  |
| **Dare  (2007)** | Thailand | Pneumonia 1890  ILI 513 | 280 | All ages | Hospitalised with pneumonia  Outpatients with ILI | Asymptomatic controls age-frequency matched to pneumonia, recruited from the same outpatient clinic with no fever, cough, sore throat or diarrhoea within past 3 days | Case | 35 (1.9) | 8 (0.4) | 10 (0.5) | 12 (0.6) | No HCoVs were associated with pneumonia  NL63 was more commonly detected in controls than pneumonia cases (OR 0.07, 95%CI 0.01-0.84, p=0.04) |
|  |  |  |  |  |  |  | ILI | 0 (0) | 0 (9.8) | 2 (0.4) | 1 (0.2) |  |
|  |  |  |  |  |  |  | Control | 1 (0.4) | 2 (0.4) | 1 (0.4) | 2 (0.7) |  |
| **Graat (2003)** | The Netherlands | 107 ARI episodes in 97 cases | 99 | >60 years | ARI, defined as sudden onset rhinorrhoea/ sneezing, sore throat/hoarseness, or dry cough for >2 day | Asymptomatic controls frequency matched for age, sex and calendar time (within 8 weeks before/after onset of case symptoms) | HCoV OC43 and 229E detected in 18 (17%) cases and 2/91 (2%) controls. | | | | | No difference between cases and controls for HCoV OC43 and 229E combined. |
| **Heimdal (2019)** | Norway | 3458 | 373 | <16 years | Hospitalised with RTI | Children admitted for elective surgery (excluding ENT surgery) without symptoms of RTIs within past 2 weeks | Case | 146 (4.2) | 101 (2.9) | 18 (0.5) | 50 (1.4) | HCoV-positive children with RTIs were more likely to have a high genome load (Ct <28) than asymptomatic controls (OR 2.59, p-0.01) |
|  |  |  |  |  |  |  | Control | 12 (3.2) | 14 (3.8) | 6 (1.6) | 7 (1.9) |  |
| **Ieven (2018)** | Europe | 3104 | 2985 | >18 years | Acute/worsening cough (<28 days duration) as main symptom, or any clinical presentation considered to be caused by LRTI | Control patient attending GP for reason other than acute respiratory illness, matched for age, gender, and time period. | HCoVs detected in 231 (7.4%) cases and 29/2063 (1.4%) controls.  Species-specific prevalence was not reported in cases or controls. | | | | | HCoVs detected significantly more frequently in cases than controls. |
| **Jain (2015)** | USA | 2179 | 521 | <18 years | Hospitalised with evidence of acute infection and an acute respiratory illness, and radiographic evidence of pneumonia | Asymptomatic elective surgical outpatients with no fever or respiratory symptoms within 14 days before or after enrolment. Same exclusion criteria as cases | HCoVs detected in 110 (5.0%) of cases and <3% of controls (exact number not reported). Species-specific prevalence was not reported in cases or controls. | | | | | Not reported. |
| **Jain (2015)** | USA | 2259 | 238 | >18 years | Hospitalised with evidence of acute infection and an acute respiratory illness, and radiographic evidence of pneumonia | Asymptomatic adults attending a general medicine clinic with no fever or respiratory symptoms within 14 days before or after enrolment. Same exclusion criteria as cases | HCoVs detected in 53 (2.3%) of cases and prevalence in controls not reported.  Species-specific prevalence was not reported in cases or controls. | | | | | Not reported. |
| **Nicol (2021)** | South Africa | 290  (464 LRTI events) | 315  (464 LRTI events) | <1 year | WHO-defined pneumonia | Incidence-density-matched (by birth date, age of presentation and site) controls with no pneumonia | Case    Control | 24 (5.6)    14 (3.0) | 11 (2.5)    11 (2.4) | 4 (0.9s)    1 (0.2) | 8 (1.9)    12 (2.6) | HCoV-OC43 associated with LRTI (OR 2.5, 95%CI 1.4-4.2) |
| **Owusu (2014)** | Ghana | 593 | 620 | >10 years | Upper respiratory illness with rapid onset of any of: cough, sneezing, runny nose and nasal congestion | No symptom of upper respiratory illness for > 8 days prior to recruitment | Case | 18 (3) | 30 (5.1) | 36 (6.1) | 5 (0.8) | HCoV-229E (OR 5.15, 95%CI 2.24-11.78), HCoV-OC43 (OR 6.16, 95%CI 1.77-21.65) associated with URTI. |
|  |  |  |  |  |  |  | Control | 3 (0.5) | 53 (8.5) | 9 (1.5) | 0 (0) |  |
| **Qin (2022)** | China | 900* | 595 | All ages | Radiographic evidence of pneumonia | Asymptomatic controls with no fever or respiratory symptoms 14 days before or after enrolment. Same exclusion criteria as cases.  Controls only recruited from 1 site (Chengdu), while cases were from 4 sites. Not time-matched to cases. | HCoVs were detected in 75 (8.3%) cases and 0 (0%) controls | | | | | Not reported |
| **Rhedin (2015)** | Sweden | 121 | 240 | <5 years | Radiographic evidence of CAP | Children attending child health units for routine visits, matched to cases on age and calendar time | Case    Control | 1 (1)    8 (3) | 1 (1)    3 (1) | 1 (1)    2 (1) | 3 (2)    16 (7) | Adjusted OR for association of HCoVs with CAP was 0.2 (95%CI 0.0-0.8). |
| **Singleton (2010)** | USA | 440 | 425 | <3 years | Hospitalised with LRTI | Children in the community with no respiratory symptoms within 14 days of enrolment    Controls not time-matched. | HCoVs detected in 25 (6%) cases and 15 (4%) controls.  Species-specific prevalence was not reported in cases or controls. | | | | | Attributable fraction among the exposed (AFE) was restricted to Year 2 of study, where 4% of cases and 4% controls were positive for HCoVs, with an AFE of 15% (95% CI -93,63) |
| **Van Elden (2004)** | The Netherlands | 261 | 243 | Not stated | URTI, LRTI, and adult patients with pneumonia | Healthy volunteers & asymptomatic bone marrow transplant recipient | HCoVs present in 28 (11%) cases and 1 (0.4%) controls. No species-specific prevalence reported in cases or controls. | | | | | Not reported |
| **Van  Gageldonk-Lafeber (2005)** | The Netherlands | 541 | 541 | All ages | GP-attended ILI or ARTI, first consultation for the episode, and not used antibiotic or antivirals in previous 2 weeks. | Asymptomatic persons attending GP practice with no respiratory complaints in previous 2 weeks, not in same household as a case, and not used antibiotic or antivirals in previous 2 weeks. | HCoVs present in 5 (3.0%) ILI, 30 (8%) ARTI, and 30 (5.5%) controls.  No species-specific prevalence reported in cases or controls. | | | | | Not reported |
| **Venter (2011)** | South Africa | 610 | 46 | <5 years | ARI | Healthy children from the same region as cases. | Cases    Controls | 11 (1.8)    0 (0) | 13 (2.1)    0 (0) | 2 (0.3)    0 (0) | 1 (0.2)    0 (0) | Not reported |

**Abbreviations**: CAP, community-acquired pneumonia; Ct, cycle threshold; ENT, Ear, Nose and Throat; HCoV, human coronaviruses; HIV, human immunodeficiency virus; ILI, influenza-like illness; OR, odds ratio; RTI, respiratory tract infections; URTI, upper respiratory tract infection; LRTI, lower respiratory tract infection; WHO, World Health Organization; CI, confidence interval

*Only included cases from the study site that recruited controls

## **Supplementary Table 3.** Prevalence of human coronaviruses and coinfections reported in included studies

| Source | | N samples tested | All HCoV N (%) | | Individual HCoV | | | | | | | | Co-infections with other respiratory viruses | | Co-infecting virus |
| --- | --- | --- | --- | --- | --- | --- | --- | --- | --- | --- | --- | --- | --- | --- | --- |
|  |  |  |  |  | **OC43**  **(N%)** | | **NL63**  **N (%)** | | **229E**  **N (%)** | | **HKU1**  **N (%)** | |  |  |  |
| Agustiningsih (2012) | | 230 | 3 (1.3) | | NR | | 3 (1.3) - grouped with 229E | | 3 (1.3) - grouped with NL63 | | NR | | N | | - |
| Ahn (2014) | | 1,528 | 35 (2.3) | | NR | | NR | | NR | | NR | | Y | | HBoV, HMPV, ADV, RSV, |
| Akagi (2021) | | 2,034 | 121 (5.9) | | 21 (1.0) | | 11 (0.5) | | 12 (0.6) | | 80 (3.9) | | Y | | RSV, Flu |
| Al-Khannaq (2016) | | 2,060 | 68 (3.3) | | NR | | 45 (2.2) | | 23 (1.1) | | NR | | N | | - |
| Al-Romaihi (2020) | | 30,946 | 1,741 (5.6) | | 840 (2.7) | | 378 (1.2) | | 188 (0.6) | | 334 (1.1) | | N | | - |
| Ali (2016) | | 817 | 21 (2.6) | | 11 (1.4) | | 4 (0.5) | | 1 (0.1) | | 5 (0.6) | | Y | | EV/RV |
| Altay-Kocak (2022) | | 1592 | 158 (9.9) | | NR | | NR | | NR | | NR | | N | | - |
| Amer (2016) | | 174 | 14 (8) | | NR | | NR | | NR | | NR | | N | | - |
| Anand (2020) | | 513 | 14 (2.7) | | NR | | NR | | NR | | NR | | Y | | RV |
| Angeles Marcos (2006) | | 198 | 5 (2.5) | | NR | | NR | | NR | | NR | | Y | | RSV, Flu A |
| Annan (2016) | | 1,713 | 110 (6.4) | | NR | | NR | | NR | | NR | | Y | | NR |
| Arden (2005) | | 840 | 16 (1.9) | | NR | | 16 (1.9) | | NT | | NT | | Y | | ADV, RV, HMPV |
| Aygun (2020) | | 422 | 8 (1.9) | | NR | | NR | | NR | | NT | | N | | - |
| Azziz-Baumgartner (2021) | | 964 | 42 (4) | | NR | | NR | | NR | | NR | | N | | - |
| Baillie (2021) | | 1,537 | 104 (6.8) | | 42 (2.7) | | 36 (2.3) | | 8 (0.5) | | 21 (1.4) | | Y | | RSV, HBoV, ADV |
| Beka (2013) | | 109 | 1 (0.9) | | NR | | NR | | NR | | NR | | N | | - |
| Bellei (2008) | | 420 | 15 (3.6) | | 10 (2.4) | | NT | | 5 (1.2) | | NT | | N | | - |
| Berce (2015) | | 278 | 14 (5) | | NR | | NR | | NR | | NR | | Y | | NR |
| Berkley (2010) | | 759 | 76 (10.0) | | 14 (1.8) | | 10 (1.3) | | 51 (6.7) | | 1 (0.1) | | Y | | RSV |
| Bimouhen (2022) | | 2,009 | 94 (4.7) | | 22 (1.1) | | 38 (1.9) | | 15 (0.7) | | 19 (1) | | N | | - |
| Bouvier (2018) | | 902 | 82 (9.1) | | 28 (3.1) | | 18 (2) | | 23 (2.5) | | 13 (1.5) | | Y | | RSV, ADV, Flu A, Flu B |
| Brini (2017) | | 372 | 80 (21.5) | | NR | | NR | | 45 (12) | | NR | | N | | - |
| Brini (2019) | | 515 | 95 (18.4) | | 8 (1.6) | | 6 (1.2) | | 42 (8.2) | | 39 (7.6) | | Y | | ADV |
| Brittain-Long (2011) | | 7,220 | 239 (3.0) | | 124 (1.6) | | 82 (1.0) | | 33 (0.4) | | NT | | N | | - |
| Cabeca (2013) | | 1,137 | 88 (7.7) | | 24 (2.1) | | 44 (3.9) | | 15 (1.3) | | 5 (0.4) | | Y | | ADV, RV |
| Calvo (2020) | | 5,131 | 205 (4.0) | | NR | | NR | | NR | | NR | | Y | | RSV, RV |
| Canducci (2008) | | 322 | 28 (8.7) | | 11 (3.4) | | 10 (3.1) | | 6 (1.9) | | 1 (0.3) | | Y | | RSV, HMPV |
| Cebey-López (2015) | | 204 | 5 (2.5) | | NR | | NR | | NR | | NT | | Y | | RSV, ADV, HBoV |
| Chen (2022) | | 1,358 | 68 (5.0) | | 9 (0.7) | | 24 (1.8) | | 4 (0.3) | | 31 (2.28) | | N | | - |
| Chen Y (2021) | | 2,057 | 67 (3.2) | | NR | | NR | | NR | | NR | | Y | | Flu A |
| Chiu (2005) | | 587 | 26 (4.4) | | 6 (1) | | 12 (2) | | 2 (0.34) | | NT | | Y | | Flu A, PIV-3 |
| Choi (2021) | | 9,589 | 463 (4.8) | | 268 (2.8) | | 139 (1.4) | | 67 (0.7) | | NT | | Y | | ADV, PIV, RSV, Flu A, Flu B, HRV, HBoV, HMPV, EV |
| Chonmaitree (2008) | | 864 | 63 (7.2) | | NR | | NT | | NR | | NT | | N | | - |
| Chow (2022) | | 14,464 | 107 (0.7) | | 6 (0.04) | | 24 (0.1) | | 6 (0.04) | | 64 (0.4) | | N | | - |
| Ciotti (2020) | | 539 | 17 (3.1) | | 6 (1.1) | | 5 (0.9) | | 3 (0.6) | | 3 (0.6) | | N | | - |
| Cui (2015) | | 1,074 | 155 (14.4) | | NR | | NR | | NR | | NR | | Y | | EV/RV, PIV |
| Dare (2007) | | 1,890 | 64 (3.4) | | 35 (1.9) | | 8 (0.4) | | 10 (0.5) | | 12 (0.6) | | Y | | RSV, HPIV, Flu, RV, ADV, HBoV |
| Das (2015) | | 254 | 7 (2.8) | | NR | | NR | | NR | | NT | | Y | | HMPV |
| De Conto (2019) | | 2,892 | 262 (9.1) | | NR | | NR | | NR | | NR | | Y | | HMPV, PIV, Flu B ADV |
| Delangue (2014) | | 564 | 28 (5) | | 28 (5), grouped with 229E | | NT | | 28 (5), grouped with OC43 | | NT | | Y | | ADV, Flu A |
| Diederen (2009) | | 242 | 19 (8) | | NR | | NT | | NR | | NT | | N | | - |
| Do (2011) | | 309 | 24 (7.7) | | 2 (0.7) | | 21 (7) | | 1 (0.3) | | NT | | Y | | Flu |
| Dos Santos Ferreira (2019) | | 150 | 6 (4) | | 4 (2.7) | | 2 (1.3) | | NT | | NT | | N | | - |
| Dyrdak (2021) | | 55,190 | 2130 (3.9) | | NR | | NR | | NR | | NR | | Y | | RSV, ADV, HBoV, RV |
| Essa (2015) | | 735 | 39 (5.3) | | 25 (3.4) | | 0 (0) | | 14 (1.9) | | NT | | N | | - |
| Etenna (2014) | | 1,041 | 61 (5.9) | | 33 (3.2) | | 12 (1.2) | | 6 (0.6) | | 10 (1.0) | | N | | - |
| Fagbo (2015) | | 2,235 | 77 (3.4) | | NR | | NR | | NR | | NT | | Y | | NR |
| Fairchok (2010) | | 318 | 48 (15.1) | | NR | | NR | | NR | | NR | | Y | | NR |
| Faye (2023) | 9,337 | | 406 (4.3) | 235 (2.5) | | 102 (1.1) | | 58 (0.6) | | 17 (0.2) | | Y | | Flu, ADV, RV | |
| Feng (2014) | | 31,348 | 393 (1.3) | | NR | | NR | | NR | | NR | | Y | | RSV, Flu, ADV, HBoV, HMPV |
| Ferreira (2019) | | 150 | 6 (4) | | 4 (2.7) | | 2 (1.3) | | NT | | NT | | N | | - |
| Fillatre (2018) | | 3,199 | 116 (3.6) | | NR | | NR | | NR | | NR | | Y | | Flu A, PIV, RSV, RV, HBoV |
| Frutos (2022) | | 9,018 | 610 (6.8) | | 323 (3.6) | | 163 (1.8) | | 86 (1.0) | | 69 (0.8) | | N | | - |
| Fu (2015) | | 1970 | 51 (2.6) | | 16 (0.8) - grouped with HKU1 | | 35 (1.8) - grouped with 229E | | 35 (1.8) - grouped with NL63 | | 16 (0.8) - grouped with OC43 | | Y | | Flu B, RSV, PIV |
| Furuse (2010) | | 411 | 3 (0.7) | | 1 (0.2) | | NR | | NR | | 2 (0.5) | | N | | - |
| Gagneur (2008) | | 244 | 50 (20.5) | | NT | | NT | | 50 (20.5) | | NT | | N | | - |
| Garbino (2006) | | 540 | 29 (5.4) | | 12 (2.2) | | 6 (1.1) | | 7 (1.3) | | 4 (0.74) | | N | | - |
| Garbino (2009) | | 522 | 30 (5.7) | | NR | | NR | | NR | | NR | | N | | - |
| Gaunt (2010) | | 11,661 | 282 (2.4) | | 111 (1) | | 75 (0.6) | | 35 (0.3) | | 61 (0.5) | | Y | | ADV, Flu A, Flu B, PIV |
| Gil (2018) | | 524 | 32 (6.1) | | 24 (4.6) | | 5 (0.9) | | 3 (0.6) | | NT | | N | | - |
| Goes (2019) | | 282 | 34 (12.1) | | 5 (1.8) | | 4 (1.4) | | 8 (2.8) | | 17 (6.0) | | Y | | RV, RSV, PIV |
| Goktas (2016) | | 845 | 51 (6.0) | | NR | | NR | | NR | | NR | | Y | | Flu A |
| Graat (2003) | | 107 | 18 (10.7) | | NR | | NR | | NT | | NT | | Y | | RV, Flu B |
| Guerrier (2013) | | 1,006 | 9 (0.9) | | NR | | NR | | NR | | NR | | Y | | RSV, RV |
| Haddadin (2021) | | 3,168 | 177 (5.6) | | 99 (3.1) | | 39 (1.2) | | 11 (0.3) | | 23 (0.7) | | N | | - |
| Hajjar (2010) | | 144 | 4 (2.8) | | NT | | 4 (2.8) | | NT | | NT | | N | | - |
| Han (2007) | | 827 | 14 (1.7) | | NT | | 14 (1.7) | | NT | | NT | | N | | - |
| Hara (2015) | | 495 | 15 (3) | | 8 (1.6) | | 5 (1.2) | | 0 (0) | | 1 (0.2) | | Y | | ADV, RSV, PIV, RV |
| Hasuwa (2020) | | 373 | 0 (0) | | 0 (0) | | NT | | 0 (0) | | NT | | N | | - |
| Hatem (2019) | | 3,207 | 0 (0) | | 0 (0) | | 0 (0) | | 0 (0) | | 0 (0) | | N | | - |
| Hautala (2020) | | 1,899 | 66 (4) | | NR | | NR | | NR | | NT | | N | | - |
| Hawkes (2021) | | 37,719 | 4,657 (12.4) | | 1,385 (3.7) | | 1,540 (4.1) | | 1,105 (2.9) | | 627 (1.7) | | N | | - |
| Heimdal (2019) | | 3,831 | 313 (9.1) | | 146 (4.2) | | 101 (2.9) | | 18 (0.5) | | 50 (1.5) | | Y | | RSV, RV, EV, HBoV, PIV, Flu |
| Heimdal (2022) | | 4,312 | 341 (8) | | NR | | NR | | NR | | NT | | Y | | Flu, HMPV, ADV, HBoV, PIV, EV, RSV, RV |
| Helou (2022) | | 100 | 6 (6) | | NR | | NR | | NR | | NR | | N | | - |
| Hoffmann (2012) | | 295 | 37 (12.5) | | NR | | NR | | NR | | NR | | Y | | HBoV, PIV, RV, |
| Hu (2014) | | 559 | 72 (12.9) | | 70 (12.5) | | NT | | NT | | NT | | Y | | RV, Flu A, RSV, HMPV, Flu B, PIV-2 |
| Huang (2013) | | 279 | 14 (5) | | 4 (1.4) | | 7 (2.5) | | 1 (0.36) | | 2 (0.7) | | Y | | NR |
| Huang, S-H (2017) | | 282 | 17 (6.03) | | NT | | 17 (6.0) | | NT | | NT | | Y | | Flu A |
| Huang, X-B (2020) | | 22,680 | 856 (7.1) | | NR | | NR | | NR | | NR | | Y | | Flu A/B, RSV, PIV, ADV, HBoV |
| Ieven (2018) | | 3,104 | 231 (7.4) | | NR | | NR | | NR | | NT | | Y | | NR |
| Jain (2015) | | 2,259 | 53 (2.3) | | NR | | NR | | NR | | NR | | N | | - |
| Jain (2015) | | 2,222 | 110 (5) | | NR | | NR | | NR | | NR | | Y | | Flu, HMPV, PIV, ADV |
| Jean (2013) | | 3,847 | 68 (1.8) | | 68 (1.8) | | NT | | NT | | NT | | Y | | ADV, RV/EV, RSV, PIV |
| Jeon (2019) | | 1362 | 31 (2.3) | | 12 (0.9) | | 8 (0.6) | | 11 (0.8) | | NT | | Y | | Flu A, RV, ADV |
| Jevsnik (2012) | | 664 | 40 (6.0) | | 7 (1.1) | | 6 (0.9) | | 6 (0.9) | | 21 (3.2) | | Y | | RV, HBoV, RSV, HMPV, ADV |
| Jin (2010) | | 645 | 19 (3.0) | | NR | | NR | | NR | | 19 (3.0) | | Y | | RSV, RV, Flu A, ADV |
| Jin (2012) | | 813 | 49 (6.0) | | NT | | 31 (3.8) | | NT | | 18 (2.2) | | Y | | RV, RSV |
| Jo (2022) | | 22,542 | 1,096 (4.9) | | 624 (2.8) | | 371 (1.7) | | 86 (0.4) | | 33 (0.2) | | Y | | RSV, RV, ADV |
| Johnstone (2008) | | 193 | 4 (2.1) | | 4 (2.1) | | 0 (0) | | 0 (0) | | NT | | N | | - |
| Kadjo (2018) | | 1059 | 40 (3.8) | | 3 (0.3) | | NT | | 37 (3.5) | | NT | | Y | | PIV, RSV |
| Kenmoe (2016) | | 347 | 20 (5.8) | | NR | | NR | | NR | | NR | | Y | | Flu B |
| Khalifa (2018) | | 515 | 95 (18.4) | | 8 (1.6) | | 6 (1.2) | | 42 (8.1) | | 39 (7.6) | | Y | | NR |
| Khamis (2012) | | 259 | 1 (0.4) | | NR | | 1 (0.4) - grouped with 229E | | 1 (0.4) - grouped with NL63 | | NR | | Y | | PIV |
| Khomenko (2021) | | 487 | 50 (10.3) | | 12 (2.5), grouped with HKU1 | | 38 (7.8), grouped with 229E | | 38 (7.8) , grouped with NL63 | | 12 (2.5), grouped with OC43 | | Y | | RV, ADV, PIV |
| Killerby (2018) | | 854,575 | 39,588 (4.6) | | 18,804 (2.2) | | 8,558 (1.0) | | 7,001 (0.8) | | 5,225 (0.6) | | N | | PIV, RSV, HMPV, ADV, RV/EV, Flu A/B |
| Kim H-C (2016) | | 477 | 34 (7.1) | | 16 (3.4) | | NR | | NR | | NT | | N | | - |
| Kim J-M (2018) | | 36,915 | 1,537 (4.2) | | NR | | NR | | NR | | NT | | N | | - |
| Kim JM (2020) | | 9010 | 364 (4) | | NR | | NT | | NR | | NT | | N | | - |
| Kim T (2021) | | 24,311 | 807 (3.3) | | 416 (1.7) | | 267 (1.1) | | 124 (0.5) | | NT | | N | | - |
| Kiyuka (2018) | | 5,573 | 75 (1.3) | | NT | | 75 (1.3) | | NT | | NT | | N | | - |
| Klivleyeva (2023) | | 4,712 | 22 (0.5) | | NR | | NR | | NR | | NR | | Y | | - |
| Koetz (2006) | | 212 | 12 (5.67) | | NT | | 12 (5.7) | | NT | | NT | | Y | | HMPV |
| Koker (2019) | | 219 | 40 (18.3) | | 19 (8.7) | | 4 (1.8) | | 10 (4.6) | | 7 (3.2) | | Y | | PIV-3, RV |
| Komabayashi (2021) | | 7,538 | 242 (3.2) | | 242 (3.2) | | NR | | NR | | NR | | Y | | ADV, PIV, EV, RSV, Flu, HPMY |
| Kong (2021) | | 4541 | 163 (3.6) | | 68 (1.5) | | 64 (1.4), grouped with HKU1 | | 32 (0.7) | | 64 (1.4), grouped with HKU1 | | Y | | PIV, EV/RV, Flu A/B, ADV, RSV, BoCV, HMPV |
| Kozak (2020) | | 5,038 | 569 (11.3) | | 285 (5.7) | | 78 (1.6) | | 127 (2.5) | | 79 (1.6) | | Y | | NR |
| Kumar (2017) | | 250 | 44 (17.6) | | 11 (4.4) | | 12 (4.8) | | 5 (2.0) | | 16 (6.4) | | N | | - |
| Kumar (2020) | | 755 | 49 (6.5) | | 16 (2.1) | | 11 (1.4) | | 11 (1.4) | | 9 (1.2) | | Y | | RV, PIV, HMPV |
| Kuribayashi (2022) | | 1,769 | 85 (4.8) | | 47 (2.7) | | 38 (2.1) | | 0 (0) | | 0 (0) | | N | | - |
| Kurskaya (2018) | | 1,560 | 13 (0.8) | | NR | | NR | | NR | | NR | | Y | | RSV, PIV |
| Kuypers (2007) | | 1,043 | 66 (6.3) | | 19 (1.8) | | 11 (1.1) | | 8 (0.8) | | 28 (2.6) | | Y | | RSV, Flu A, PIV, HMPV, ADV |
| Lambert (2007) | | 730 | 1 (0.14) | | NT | | 1 (0.1) | | NT | | NT | | Y | | HMPV, PIV |
| Lau (2006) | | 4,181 | 87 (2.1) | | 53 (1.3) | | 17 (0.4) | | 4 (0.1) | | 13 (0.3) | | N | | - |
| Le (2020) | | 348 | 2 (0.6) | | NT | | NT | | 1 (0.3) | | 1 (0.3) | | Y | | EV |
| Lee (2013) | | 1,985 | 50 (2.5) | | NT | | NT | | NT | | 50 (2.5) | | N | | - |
| Leli (2021) | | 572 | 11 (2) | | 2 (0.4) | | 1 (0.2) | | 4 (0.7) | | 4 (0.7) | | N | | - |
| Li (2018) | | 2,768 | 120 (4.3) | | NR | | NR | | NR | | NR | | N | | - |
| Li (2021) | | 397 | 24 (6) | | 4 (1) | | 8 (2) | | 6 (1.5) | | 6 (1.5) | | Y | | Flu A, ADV, PIV |
| Li Y-T J (2019) | | 659 | 15 (2.3) | | NR | | NR | | NR | | NR | | N | | - |
| Litwin (2014) | | 939 | 80 (8.5) | | 14 (1.5) | | 34 (3.6) | | 2 (0.2) | | 30 (3.2) | | Y | | RSV, HMPV |
| Liu, P (2017) | | 607 | 22 (3.6) | | NR | | NR | | NR | | NR | | N | | - |
| Liu, T (2017) | | 3,298 | 78 (2.4) | | 36 (1.0) | | 6 (0.2) | | 2 (0.01) | | 34 (1.0) | | Y | | NR |
| Liu, WK(2014) | | 4,242 | 231 (5.5) | | 138 (3.3) | | 39 (0.9) | | 37 (0.9) | | 17 (0.4) | | Y | | NR |
| Low (2022) | | 23,306 | 819 (3.5) | | NR | | NR | | NR | | NR | | Y | | NR |
| Lu (2013) | | 596 | 4 (0.7) | | NR | | NT | | NR | | NT | | Y | | Flu B |
| Malhotra (2016) | | 155 | 12 (7.7) | | 5 (3.2) | | 1 (0.6) | | 1 (0.6) | | 5 (3.2) | | Y | | ADV, RV |
| Matienzo (2020) | | 1,315 | 41 (3.04) | | NR | | NR | | NR | | NR | | N | | - |
| Matoba (2015) | | 4,342 | 332 (7.6) | | 78 (1.8) | | 133 (3.1) | | 38 (0.9) | | 83 (1.9) | | Y | | EV, HPIV, ADV, RV, HMPV, Flu A/B, RSV |
| Matsuno (2019) | | 279 | 27 (9.7) | | 11 (3.9) | | 2 (0.7) | | 9 (3.2) | | 5 (1.8) | | Y | | RV |
| Mohammadi (2020) | | 138 | 33 (23.9) | | NT | | 33 (23.9) | | NT | | NT | | N | | - |
| Monto (2020) | | 7,992 | 993 (12.7) | | 387 (3.8) | | 323 (4.0) | | 149 (1.9) | | 193 (2.4) | | N | | - |
| Nascimento-Carvalho (2018) | | 774 | 64 (8.3) | | 43 (5.6) | | 16 (2.1) | | 13 (1.7) | | NT | | Y | | NR |
| Nguyen (2016) | | 435 | 33 (7.6) | | NR | | NR | | NR | | NR | | N | | - |
| Nickbakhsh (2016) | | 44,230 | 1,339 (4.1) | | NR | | NR | | NR | | NR | | Y | | RSV, ADV, PIV-3 |
| Nickbakhsh (2020) | | 74,519 | 2,958 (4) | | NR | | NR | | NR | | NR | | Y | | RSV, ADV, PIV-3 |
| Nicol (2021) | | 896 | 46 (10.2) | | 24 (5.6) | | 11 (2.5) | | 4 (0.9) | | 8 (1.9) | | Y | | RSV, Flu A/B, PIV, ADV |
| Noyola (2019) | | 5,392 | 732 (13.6) | | NR | | NR | | NR | | NR | | Y | | HMPV, RSV, PIV, RV, Flu |
| Nunes (2014) | | 1,460 | 156 (10.7) | | 97 (6.6) | | 33 (2.3) | | 4 (0.3) | | 22 (1.5) | | Y | | WU PyV, KI PyV, RV, PIV, HBoV, HMPV, RSV |
| Nyiro (2018) | | 5,647 | 387 (6.9) | | NR | | NR | | NR | | NR | | Y | | NR |
| Ortiz-Hearnandez (2019) | | 1,486 | 58 (4) | | NR | | NR | | NR | | NR | | Y | | ADV, Flu A/B |
| Otieno (2020) | | 7957 | 314 (3.9) | | 129 (1.6) | | 74 (0.9) | | 99 (1.2) | | NT | | N | | - |
| Ottogalli (2020) | | 631 | 19 (3.01) | | 14 (2.2) | | NT | | 5 (0.8) | | NT | | N | | - |
| Owusu (2014) | | 593 | 81 (13.7) | | 18 (3.0) | | 30 (5.1) | | 36 (6.1) | | 5 (0.8) | | N | | - |
| Price (2019) | | 33,652 | 455 (6.8) | | NR | | NR | | NR | | NR | | Y | | Flu A, HMPV |
| Qin (2021) | | 1,674 | 138 (8.3) | | NR | | NR | | NR | | NR | | N | | - |
| Qu (2015) | | 954 | 8 (0.8) | | OC43/HKU1 (0.4) | | NL63/229E 4 (0.4) | | NL63/229E 4 (0.4) | | OC43/HKU1 (0.4) | | Y | | Flu A, ADV |
| Radin (2014) | | 1,444 | 66 (4.6) | | 44 (3.1) | | 8 (0.6) | | 10 (0.7) | | NT | | Y | | FluA, RV, RSV |
| Razuri (2015) | | 172 | 11 (6.4) | | 0 (0) | | 2 (1.16) | | 1 (0.6) | | 8 (4.7) | | N | | - |
| Refay (2022) | | 259 | 3 (1.2) | | 0 (0) | | 2 (0.8) | | 0 (0) | | 1 (0.4) | | Y | | Flu B |
| Regamey (2008) | | 112 | 20 (17.8) | | 7 (6.3) | | 9 (7.1) | | 3 (2.7) | | 1 (0.9) | | Y | | RV, HMPV, HBoV |
| Reina (2014) | | 686 | 48 (7) | | 24 (3.5) | | 14 (2) | | 10 (1.5) | | NT | | Y | | RSV, HBoV, RV |
| Ren (2009) | | 5,808 | 65 (1.1) | | NR | | NR | | NR | | NR | | Y | | Flu A, PIV, EV, RV |
| Ren (2011) | | 8,396 | 87 (1) | | 50 (0.6) | | 8 (0.1) | | 15 (0.2) | | 14 (0.2) | | N | | - |
| Rhedin (2015) | | 361 | 35 (9.7) | | 9 (2.5) | | 4 (1.1) | | 3 (0.8) | | 19 (5.3) | | Y | | RSV |
| Sarna (2017) | | 8,100 | 157 (1.9) | | 51 (0.6) | | 56 (0.7) | | 14 (0.2) | | 34 (0.4) | | Y | | RV, PIV |
| Sentilhes (2013) | | 292 | 7 (4) | | NR | | NR | | NR | | NR | | N | | - |
| Seo (2014) | | 3865 | 13 (0.3) | | 13 (0.3) | | NT | | 0 (0) | | NT | | N | | - |
| Shah (2022) | | 82,768 | 5,204 (6.3) | | 2,056 (2.5) | | 1,519 (1.8) | | 732 (0.9) | | 962 (1.2) | | Y | | Flu, RV, RSV, ADV, PIV |
| Shan (2019) | | 445 | 36 (8) | | NR | | NR | | NR | | NR | | Y | | Flu, ADV, EV |
| Shi (2023) | | 10,396 | 280 (2.7) | | NR | | NR | | NR | | NR | | N | | - |
| Singleton (2010) | | 440 | 25 (5.7) | | NR | | NR | | NR | | NR | | N | | - |
| Sipulwa (2016) | | 417 | 35 (8.4) | | 12 (2.9) | | 10 (2.4) | | 4 (1) | | 9 (2.2) | | N | | - |
| Sonawane (2019) | | 100 | 3 (0.03) | | 1 (0.01) | | 1 (0.01) | | 0 (0) | | 1 (0.01) | | N | | - |
| Sonmezer (2023) | | 7,861 | 212 (2.7) | | 64 (0.8) | | 72 (0.9) | | 63 (0.8) | | NT | | Y | | ADV, RV, RSV, Flu B, HBoV, PIV, HMPV |
| Srinivasan (2013) | | 253 | 22 (8.7) | | 13 (5.1) | | 9 (3.6) | | 0 (0) | | 2 (0.8) | | Y | | RSV, RV, HBoV |
| Sung (2009) | | 475 | 18 (3.8) | | 16 (3.4) | | NT | | 2 (0.4) | | NT | | N | | - |
| Suzuki (2012) | | 819 | 3 (0.4) | | 1 (0.1) | | 2 (0.2) | | NT | | NT | | Y | | NR |
| Talbot (2009a) | | 1,055 | 19 (1.8) | | 3 (0.3) | | 12 (1.2) | | 0 (0) | | 4 (0.4) | | Y | | RSV, PIV, HMPV |
| Talbot (2009b) | | 1,854 | 92 (4.9) | | 54 (2.9) | | 30 (1.6) | | 8 (0.4) | | NT | | Y | | NR |
| Tao (2018) | | 320 | 8 (2.5) | | NR | | NR | | NR | | NR | | N | | - |
| Thaemboonlers (2005) | | 226 | 10 (4.4) | | 0.88 (2) | | NT | | 3.54 (8) | | NT | | N | | - |
| Trombetta (2016) | | 755 | 34 (4.5) | | NR | | NR | | NR | | NR | | Y | | RSV, HRV |
| Tsagarakis (2017) | | 656 | 36 (8.3) | | 13 (2.0) | | 9 (1.4) | | 4 (0.6) | | 10 (1.5) | | Y | | HRV, AdV, PIV, Flu, RSV |
| Tuz (2022) | | 2,606 | 98 (3.8) | | 53 (2) | | 32 (1.3) | | 13 (0.5) | | NT | | Y | | NR |
| Uddin (2018) | | 4,223 | 282 (6.7) | | 103 (2.4) | | 70 (1.7) | | 19 (0.5) | | 78 (1.8) | | Y | | HRV, HBoV, RSV |
| Vabret (2008) | | 1,002 | 86 (8.6) | | 24 (2.4) | | 27 (2.7) | | 1 (0.1) | | 34 (0.34) | | N | | - |
| van der Zalm (2009) | | 668 | 51 (7.6) | | NR | | NR | | NR | | NT | | Y | | RV, RSV |
| Van Elden (2004) | | 261 | 28 (11) | | NR | | NR | | NT | | NT | | N | | - |
| van Gageldonk-Lafeber (2005) | | 1,082 | 56 (0.6) | | NR | | NR | | NR | | NR | | N | | - |
| Varghese (2018) | | 2,582 | 212 (8.2) | | 85 (3.3) | | 77 (3.0) | | 20 (0.8) | | 34 (1.3) | | Y | | ADV, Flu A, RSV, EV |
| Venter (2011) | | 610 | 27 (4.4) | | 11 (1.8) | | 13 (2.1) | | 2 (0.3) | | 1 (0.2) | | Y | | PIV |
| Visseaux (2017) | | 7,196 | 260 (3.6) | | NT | | NR | | NT | | NR | | Y | | NR |
| Wansaula (2015) | | 332 | 16 (5.2) | | NR | | NR | | NR | | NR | | N | | - |
| Wertheim (2015) | | 1,222 | 23 (1.9) | | 9 (0.7) | | 0 (0) | | 14 (1.1) | | 0(0) | | N | | - |
| Wong-Chew (2017) | | 1404 | 26 (1.86) | | 9 (0.64) | | 11 (0.78) | | 4 (0.28) | | 2 (0.1) | | Y | | RSV |
| Woo (2012) | | 6,272 | 98 (1.6) | | 61 (1.0) | | 19 (0.3) | | 5 (0.1) | | 13 (0.2) | | N | | - |
| Xin (2012) | | 878 | 8 (0.91) | | NR | | 8 (0.9) | | NR | | NR | | N | | - |
| Xu (2021) | | 28,369 | 637 (5) | | NR | | NR | | NR | | NT | | N | | - |
| Ye (2017) | | 967 | 20 (2.1) | | NR | | NR | | NR | | NR | | Y | | Flu, PIV, RV, HMPV |
| Ye (2023) | | 15,677 | 321 (2.0) | | 130 (0.8) | | 123 (0.8) | | 60 (0.4) | | 8 (0.1) | | N | | - |
| Yew (2019) | | 397 | 8 (2.0) | | 5 (1.3) | | 2 (0.5) | | 0 (0) | | 1 (0.3) | | Y | | PIV-1 |
| Yip (2016) | | 8,275 | 77 (0.9) | | 48 (0.6) | | 6 (0.1) | | 12 (0.1) | | 11 (0.1) | | N | | - |
| Yu (2012) | | 416 | 49 (11.8) | | 4 (1.0) | | 2 (0.5) | | 39 (9.4) | | 4 (1.0) | | Y | | NR |
| Zeng (2018) | | 11,399 | 489 (4.3) | | 346 (3) | | 60 (0.5) | | 65 (0.6) | | 38 (0.3) | | Y | | Flu A/B, RSV, HPIV, ADV, EV, HBoV, HMPV, RV |
| Zhang (2012) | | 165 | 10 (6.1) | | NR | | NR | | NR | | NR | | N | | Y |
| Zhang, D (2014) | | 14,237 | 351 (2.5) | | NR | | NR | | NR | | NR | | Y | | Flu, RSV, PIV, RV, HMPV, ADV, HBoV |
| Zhang, S-F (2018) | | 13,048 | 294 (2.3) | | 117 (0.9) | | 44 (0.3) | | 44 (0.3) | | 23 (0.2) | | Y | | Flu, RSV, PIV, RV, HMPV, ADV, HBoV |
| Zhang, Y (2021) | | 3,660 | 144 (3.9) | | 38 (1.0) | | 62 (1.7) | | 8 (0.2) | | 38 (1.0) | | N | | - |
| Zhao (2019) | | 700 | 75 (10.7) | | NR | | NR | | NR | | NR | | N | | - |
| Zhu (2021) | | 2,721 | 73 (2.7) | | 36 (1.3) | | 4 (0.15) | | 15 (0.6) | | 18 (0.7) | |  | | N |

**Abbreviations**: HCoV, human coronaviruses; NR, not reported; NT, not tested; ADV, adenovirus; EV, enterovirus; Flu, influenza; HBoV, human bocavirus; PIV, parainfluenza viruses; PyV, polyomavirus; HRV, human rhinovirus; RSV, respiratory syncytial virus

## **Supplementary Table 4.** Prevalence of human coronaviruses reported in included studies

|  | |  | |  | |  | Incidence rates | | | | |
| --- | --- | --- | --- | --- | --- | --- | --- | --- | --- | --- | --- |
|  | **Age group** | | **Setting** | | **All HCoVs** | | | **OC43** | **NL63** | **229E** | **HKU1** |
| Ali (2016) | Paediatric | | Community | | NR | | | 0.73/CY | NT | NT | NT |
| Fairchok (2010) | Paediatric | | Community | | 0.433/ CY | | | NR | NR | NR | NR |
| Frutos (2022) | Paediatric | | Primary | | 61.1/1000 PY | | | NR | NR | NR | NR |
| Monto (2020) | All | | Community | | Incidence rates per 100 PY reported by age and calendar year (exact values not provided) | | | | | | |
| Sarna (2017) | Paediatric | | Community | | NR | | | 27.3/100 CY | 26.0/100 CY | 5.8/100 CY | 18.2/100 CY |
| Talbot (2009B) | Paediatric | | Primary | | 11.4/1000 CY | | | NR | NR | NR | NT |
| Uddin (2018) | Paediatric | | Community | | 255.6/ 1000 PY | | | 87.2/1000 PY | 59.6/1000 PY | 16.4/1000 PY | 65.6/1000 PY |

Abbreviations: HCoV, human coronaviruses; CY, child-year; PY, person-year; NR, not reported; NT, not tested

# **Appendix**

## **Appendix 1:** Search Terms

CINAHAL

| ***Database*** | *Search terms (no filters or limits used)* |
| --- | --- |
| ***CINAHAL*** | ti coronavirus or ab coronavirus or ti HCoV or ab HCoV or ti HCoV-nl63 or ab HCoV-nl63 or ti HCoV-hku1 or ab HCoV-hku1 or ti HCoV-oc43 or ab HCoV-oc43 or ti HCoV-229e or ab HCoV-229e or ti coronavirus nl63 or ab coronavirus nl63 or ti coronavirus oc43 or ab coronavirus oc43 or ti coronavirus hku1 or ab coronavirus hku1 or ti coronavirus 229e or ab coronavirus 229e and tx humans or people and ti epidemiological study or ab epidemiological study or ti ( epidemiology or incidence or prevalence or occurrence ) or ab ( epidemiology or incidence or prevalence or occurrence ) or ti seasonal or ab seasonal or ti seasonality or ab seasonality not ti sars-cov-1 or ab sars-cov-1 or ti ( sars-cov-2 or covid-19 or 2019-ncov or covid 19 ) or ab ( sars-cov-2 or covid-19 or 2019-ncov or covid 19 ) or ti ( mers or middle east respiratory syndrome or mers-cov ) or ab ( mers or middle east respiratory syndrome or mers-cov ) |

EMBASE

| ***Database*** | *Search terms (no filters or limits used)* |
| --- | --- |
| ***EMBASE*** | coronavirus.ti,ab. or HCoV.mp. or coronavirus nl63.ti,ab. or coronavirus oc43.ti,ab.  coronavirus hku1.ti,ab. or coronavirus 229e.ti,ab. or HCoV-229e.ti,ab. or HCoV-hku1.ti,ab.  HCoV-oc43.ti,ab. or HCoV-nl63.ti,ab. and exp respiratory tract diseases/ or exp pneumonia/ or exp respiratory tract infections/ or lrti.mp. or rti.mp. or acute respiratory infection.mp.  and epidemiology/ or epidemiolog*.ti,ab. or exp epidemiologic studies/ or season*.ti,ab. and exp/ humans not (sars or sars-2-cov or covid-19 or mers or pandemic or rhinovirus or cmv or rotavirus).ti,ab. |
|  | |

MEDLINE

| ***Database*** | *Search terms (no filters or limits used)* |
| --- | --- |
| ***MEDLINE*** | coronavirus.ti,ab. or HCoV.mp. or coronavirus nl63.ti,ab. or coronavirus oc43.ti,ab.  coronavirus hku1.ti,ab. or coronavirus 229e.ti,ab. or HCoV-229e.ti,ab. or HCoV-hku1.ti,ab.  HCoV-oc43.ti,ab. or HCoV-nl63.ti,ab. and exp respiratory tract diseases/ or exp pneumonia/ or exp respiratory tract infections/ or lrti.mp. or rti.mp. or acute respiratory infection.mp.  and epidemiology/ or epidemiolog*.ti,ab. or exp epidemiologic studies/ or season*.ti,ab. and exp/ humans not (sars or sars-2-cov or covid-19 or mers or pandemic or rhinovirus or cmv or rotavirus).ti,ab. |

WEB OF SCIENCE

| ***Database*** | *Search terms (reviewed articles only)* |
| --- | --- |
| ***WoS*** | (coronavirus OR HCoV OR coronavirus NL63 OR coronavirus OC43 OR coronavirus HKU1 OR coronavirus 229E OR HCoV-229e OR HCoV-hku1 OR HCoV-oc43 OR HCoV-nl63) AND (respiratory infection OR acute respiratory infection OR LRTI OR RTI OR pneumonia OR  respiratory tract disease) AND (epidemiology  OR  epidemiologic studies OR season OR seasonality) AND Humans NOT (sars OR sars-2-cov OR covid-19 OR covid 19 OR mers OR pandemic OR wuhan) |

SCOPUS

| ***Database*** | *Search terms (no filters or limits used)* |
| --- | --- |
| ***SCOPUS*** | (title-abs-key (coronavirus) or title-abs-key (HCoV)  or  title-abs-key (HCoV-229e) or title-abs-key (HCoV-hku1) and title-abs-key ( HCoV-oc43 )  or  title-abs-key ( HCoV-nl63 ) or title-abs-key (human and coronavirus ) and title-abs-key (respiratory and diseases  or  respiratory  and problem  or  respiratory  and distress  or  pneumonia or respiratory and tract and infections  or  acute  and respiratory  and infection ) and title-abs-key (epidemiological  and study  or  epidemiology  or  incidence  or prevalence  or occurance  or  seasonal  or  seasonality) and not title (sars-cov-1  or  sars-cov-2  or  sars-2-cov  or  covid-19  or 2019-ncov  or  covid  19  or  mers  or  middle  and east  and respiratory  and syndrome  or mers-cov  or  sars  or  covid-19)) |

## **Appendix 2.** Risk of Bias

### **Case series**

|  | 1. Were there clear criteria for inclusion in the case series? | 2. Was the condition measured in a standard, reliable way for all participants included in the case series? | 3. Were valid methods used for identification of the condition for all participants included in the case series? | "4. Did the case series have consecutive inclusion of participants?" | "5. Did the case series have complete inclusion of participants?" | 6. Was there clear reporting of the demographics of the participants in the study? | 7. Was there clear reporting of clinical information of the participants? | 8. Were the outcomes or follow up results of cases clearly reported? | 9. Was there clear reporting of the presenting site(s)/clinic(s) demographic information? | 10. Was statistical analysis appropriate? | Overall Risk of Bias of study (low, medium or high) |  |
| --- | --- | --- | --- | --- | --- | --- | --- | --- | --- | --- | --- | --- |
| Agustiningsih (2012) | Y | Y | Y | Y | Y | Y | N | Y | N | Y | Medium | |
| Ahn (2014) | Y | Y | Y | Y | Y | Y | Y | Y | Y | Y | Low | |
| Akagi (2021) | Y | Y | Y | Y | Y | Y | Y | Y | U | Y | Low | |
| Al-Khannaq (2016) | Y | Y | Y | Y | Y | Y | Y | Y | Y | Y | Low | |
| Al-Romaihi (2020) | Y | Y | Y | Y | Y | Y | Y | Y | N | Y | Low | |
| Altay-Kocak (2022) | Y | Y | Y | Y | Y | Y | N | Y | U | Y | Low | |
| Amer (2016) | Y | Y | Y | Y | Y | Y | Y | Y | Y | Y | Low | |
| Anand (2020) | N | Y | Y | Y | Y | Y | N | Y | Y | Y | Medium | |
| Angeles Marcos (2006) | Y | Y | Y | Y | Y | Y | Y | Y | U | Y | Low | |
| Annan (2016) | Y | Y | Y | Y | Y | Y | Y | Y | Y | Y | Low | |
| Arden (2005) | Y | Y | Y | Y | Y | Y | Y | Y | Y | Y | Low | |
| Aygun (2020) | Y | Y | Y | Y | Y | Y | Y | Y | Y | Y | Low | |
| Beka (2013) | U | Y | Y | Y | Y | N | N | Y | N | Y | Medium | |
| Bellei (2008) | Y | Y | Y | Y | Y | N | N | Y | Y | Y | Low | |
| Berce (2015) | Y | Y | Y | Y | Y | Y | Y | Y | Y | Y | Low | |
| Bimouhen (2022) | Y | Y | Y | Y | Y | Y | Y | Y | Y | Y | Low | |
| Bouvier (2018) | Y | Y | Y | Y | Y | Y | Y | Y | Y | Y | Low | |
| Brini (2017) | Y | Y | Y | Y | Y | Y | Y | Y | Y | Y | Low | |
| Brini (2019) | Y | Y | Y | Y | Y | Y | Y | Y | Y | Y | Low | |
| Brini Khalifa (2018) | Y | Y | Y | Y | Y | Y | Y | Y | Y | Y | Low | |
| Brittain-Long (2011) | Y | Y | Y | Y | Y | Y | Y | Y | Y | Y | Low | |
| Cabeca (2013) | Y | Y | Y | Y | Y | N | Y | Y | Y | Y | Low | |
| Calvo (2020) | Y | Y | Y | Y | Y | Y | Y | Y | Y | Y | Low | |
| Canducci (2008) | Y | Y | Y | Y | Y | Y | Y | Y | Y | Y | Low | |
| Chen (2022) | Y | U | U | Y | Y | N | N | Y | U | Y | Medium | |
| Chen Y (2021) | Y | Y | Y | Y | Y | Y | Y | Y | Y | Y | Low | |
| Chiu (2005) | Y | Y | Y | Y | Y | Y | Y | Y | Y | Y | Low | |
| Choi (2021) | Y | Y | Y | Y | Y | Y | Y | Y | Y | Y | Low | |
| Chonmaitree (2008) | Y | Y | Y | Y | Y | Y | Y | Y | Y | Y | Low | |
| Chow (2022) | Y | Y | Y | Y | Y | Y | Y | Y | Y | Y | Low | |
| Ciotti (2020) | Y | Y | Y | Y | Y | Y | Y | Y | Y | Y | Low | |
| Cui (2015) | Y | Y | Y | Y | Y | Y | Y | Y | Y | Y | Low | |
| Das (2015) | Y | Y | Y | Y | Y | Y | Y | Y | Y | Y | Low | |
| De Conto (2019) | Y | Y | Y | Y | Y | U | Y | Y | Y | Y | Low | |
| Delangue (2014) | Y | Y | Y | Y | Y | Y | Y | Y | N | Y | Low | |
| Diederen (2009) | Y | Y | Y | Y | Y | Y | Y | Y | Y | Y | Low | |
| Do (2011) | Y | Y | Y | Y | Y | Y | Y | Y | Y | Y | Low | |
| Dos Santos Ferreira (2019) | Y | Y | Y | Y | Y | Y | Y | Y | Y | Y | Low | |
| Dyrdak (2021) | Y | Y | Y | Y | Y | U | N | Y | U | Y | Low | |
| Essa (2015) | Y | Y | Y | Y | Y | U | Y | Y | Y | Y | Low | |
| Etenna (2014) | Y | Y | Y | Y | Y | N | Y | Y | Y | Y | Low | |
| Fagbo (2015) | Y | Y | Y | Y | Y | U | Y | Y | Y | Y | Low | |
| Faye (2023) | Y | Y | Y | Y | Y | Y | Y | Y | N | Y | Low | |
| Feng (2014) | Y | Y | Y | Y | Y | Y | Y | Y | Y | Y | Low | |
| Ferreira (2019) | N | Y | Y | U | Y | U | Y | Y | Y | Y | Medium | |
| Fillatre (2018) | Y | Y | Y | Y | Y | Y | Y | Y | Y | Y | Low | |
| Frutos (2022) | Y | Y | Y | Y | Y | Y | Y | Y | Y | Y | Low | |
| Fu (2015) | Y | Y | Y | Y | Y | Y | Y | Y | Y | Y | Low | |
| Furuse (2010) | Y | Y | Y | N | Y | Y | N | Y | Y | Y | Low | |
| Gagneur (2008) | N | Y | Y | Y | Y | Y | Y | Y | U | Y | Low | |
| Garbino (2006) | Y | Y | Y | Y | Y | Y | Y | Y | Y | Y | Low | |
| Gaunt (2010) | N | Y | Y | Y | Y | Y | Y | Y | Y | Y | Low | |
| Gil (2018) | Y | Y | Y | Y | Y | Y | Y | Y | Y | Y | Low | |
| Goes (2019) | Y | Y | Y | Y | Y | Y | Y | Y | Y | Y | Low | |
| Goktas (2016) | U | Y | Y | Y | Y | Y | N | Y | N | Y | High | |
| Gratt (2003) | Y | Y | Y | Y | Y | Y | U | Y | U | Y | Low | |
| Guerrier (2013) | Y | Y | Y | Y | Y | Y | Y | Y | Y | Y | Low | |
| Haddadin (2021) | Y | Y | Y | Y | Y | Y | Y | Y | N | Y | Low | |
| Hajjar (2010) | Y | Y | Y | Y | Y | Y | N | Y | N | Y | Medium | |
| Han (2007) | Y | Y | Y | Y | Y | N | Y | Y | Y | Y | Low | |
| Hara (2015) | Y | Y | Y | Y | Y | Y | Y | Y | Y | Y | Low | |
| Hasuwa (2020) | Y | Y | Y | Y | Y | Y | Y | Y | Y | Y | Low | |
| Hatem (2019) | Y | Y | Y | Y | Y | Y | U | Y | Y | Y | Low | |
| Hawkes (2021) | Y | Y | Y | N | Y | N | N | Y | N | Y | High | |
| Heimdal (2022) | Y | Y | Y | Y | Y | U | Y | Y | Y | Y | Low | |
| Helou (2022) | Y | Y | Y | Y | Y | N | N | Y | N | Y | Medium | |
| Hoffmann (2012) | Y | Y | Y | Y | Y | Y | N | Y | Y | Y | Low | |
| Hu (2014) | Y | Y | Y | Y | Y | Y | U | Y | Y | Y | Low | |
| Huang (2013) | Y | Y | Y | Y | Y | Y | Y | Y | N | Y | Low | |
| Huang, S-H (2017) | Y | Y | Y | Y | Y | Y | Y | Y | U | Y | Low | |
| Huang, X-B (2020) | Y | Y | Y | Y | Y | Y | Y | Y | Y | Y | Low | |
| Jain (2015) | Y | Y | Y | Y | Y | Y | Y | Y | Y | Y | Low | |
| Jain (2016) | Y | Y | Y | Y | Y | Y | Y | Y | Y | Y | Low | |
| Jeon (2019) | Y | Y | Y | Y | Y | Y | Y | Y | Y | Y | Low | |
| Jevsnik (2012) | Y | Y | Y | Y | Y | Y | Y | Y | Y | Y | Low | |
| Jin, Yu (2012) | Y | Y | Y | Y | Y | Y | Y | Y | Y | Y | Low | |
| Jin, Yu (2010) | Y | Y | Y | Y | Y | Y | Y | Y | U | Y | Low | |
| Jo (2022) | Y | Y | Y | Y | Y | Y | Y | Y | Y | Y | Low | |
| Johnstone (2008) | Y | Y | Y | Y | Y | Y | Y | Y | Y | Y | Low | |
| Kadjo (2018) | Y | Y | Y | Y | Y | Y | Y | Y | Y | Y | Low | |
| Kenmoe (2016) | Y | Y | Y | Y | Y | Y | Y | Y | U | Y | Low | |
| Khamis (2012) | Y | Y | Y | Y | Y | Y | Y | Y | Y | Y | Low | |
| Khomenko (2021) | N | Y | Y | Y | Y | Y | Y | Y | U | Y | Low | |
| Killerby (2018) | Y | Y | Y | Y | Y | Y | Y | Y | Y | Y | Low | |
| Kim T (2021) | Y | Y | Y | Y | Y | Y | Y | Y | U | Y | Low | |
| Kim, H-C (2016) | Y | Y | Y | Y | Y | Y | N | U | Y | Y | Low | |
| Kim, J-M (2018) | N | N | Y | Y | Y | N | N | Y | N | Y | High | |
| Kim, JM (2020) | Y | Y | Y | Y | Y | N | U | N | Y | Y | Medium | |
| Kiyuka (2018) | Y | Y | Y | Y | Y | Y | U | Y | Y | Y | Low | |
| Klivleyeva (2023) | Y | Y | Y | Y | Y | N | N | Y | N | Y | Medium | |
| Koetz (2006) | Y | Y | Y | Y | Y | Y | Y | Y | Y | Y | Low | |
| Koker (2019) | Y | Y | Y | Y | Y | Y | Y | Y | Y | Y | Low | |
| Komabayashi (2021) | N | Y | U | Y | Y | N | N | Y | Y | Y | Medium | |
| Kong (2021) | Y | Y | Y | Y | Y | N | Y | Y | Y | Y | Low | |
| Kozak (2020) | Y | Y | Y | Y | Y | Y | Y | Y | Y | Y | Low | |
| Kuribayashi (2022) | Y | Y | Y | Y | Y | Y | Y | Y | U | Y | Low | |
| Kurskaya (2018) | Y | Y | Y | Y | Y | Y | U | Y | Y | Y | Low | |
| Kuypers (2007) | Y | Y | Y | Y | U | Y | Y | Y | Y | Y | Low | |
| Lambert (2007) | Y | Y | Y | Y | Y | Y | Y | Y | Y | Y | Low | |
| Lau (2006) | Y | Y | Y | Y | Y | Y | Y | Y | Y | Y | Low | |
| Le (2020) | Y | Y | Y | Y | Y | U | N | Y | U | Y | Medium | |
| Lee (2013) | Y | Y | Y | Y | Y | N | N | Y | N | Y | Medium | |
| Leli (2021) | Y | Y | Y | N | Y | Y | N | U | Y | Y | Medium | |
| Li (2018) | Y | Y | U | Y | Y | Y | U | Y | Y | Y | Low | |
| Li (2021) | Y | Y | Y | Y | Y | Y | Y | Y | Y | Y | Low | |
| Li Y-T J (2019) | Y | Y | Y | Y | Y | Y | Y | Y | Y | Y | Low | |
| Litwin (2014) | U | Y | Y | Y | Y | Y | Y | Y | N | Y | Low | |
| Liu, Peilin (2017) | Y | Y | Y | Y | Y | Y | Y | Y | Y | Y | Low | |
| Liu, Ti (2017) | Y | Y | Y | Y | Y | Y | Y | Y | Y | Y | Low | |
| Liu, Wen Kuan (2014) | Y | Y | Y | Y | Y | U | N | Y | N | Y | Medium | |
| Low (2022) | Y | Y | Y | Y | Y | Y | Y | Y | Y | Y | Low | |
| Lu (2013) | Y | Y | Y | Y | Y | Y | Y | Y | Y | Y | Low | |
| Malhotra (2016) | Y | Y | Y | Y | Y | Y | N | Y | Y | Y | Low | |
| Matienzo (2020) | Y | Y | Y | Y | Y | Y | Y | Y | Y | Y | Low | |
| Matoba (2015) | U | U | Y | Y | Y | N | N | Y | N | Y | High | |
| Matsuno (2019) | Y | Y | Y | Y | Y | Y | Y | Y | U | Y | Low | |
| Mohammadi (2020) | N | U | Y | Y | Y | N | N | Y | N | Y | High | |
| Nascimento-Carvalho (2018) | Y | Y | Y | Y | Y | Y | Y | Y | Y | Y | Low | |
| Nguyen (2016) | Y | Y | Y | Y | Y | Y | Y | Y | U | Y | Low | |
| Nickbakhsh (2020) | Y | Y | Y | Y | Y | Y | Y | Y | Y | Y | Low | |
| Nickbakhsh (2016) | Y | Y | Y | Y | Y | Y | Y | Y | Y | Y | Low | |
| Noyola (2019) | Y | Y | Y | Y | Y | Y | Y | Y | Y | Y | Low | |
| Nunes (2014) | Y | Y | Y | N | Y | Y | Y | Y | Y | Y | Low | |
| Nyiro (2018) | Y | Y | Y | Y | Y | Y | Y | Y | Y | Y | Low | |
| Ortiz-Hearnandez (2019) | Y | Y | Y | Y | Y | Y | Y | Y | Y | Y | Low | |
| Otieno (2020) | Y | Y | U | Y | Y | N | Y | Y | Y | Y | Low | |
| Ottogalli (2020) | U | Y | Y | N | N | N | Y | Y | Y | Y | Medium | |
| Price (2019) | N | N | Y | N | Y | Y | N | Y | Y | Y | High | |
| Qin (2022) | Y | Y | Y | Y | Y | Y | Y | Y | Y | Y | Low | |
| Qu (2015) | Y | Y | Y | Y | Y | Y | Y | N | Y | Y | Low | |
| Radin (2014) | Y | Y | Y | Y | Y | Y | Y | Y | Y | Y | Low | |
| Refay (2022) | Y | Y | Y | Y | Y | U | N | Y | N | Y | Low | |
| Reina (2014) | N | U | Y | Y | Y | N | Y | N | Y | Y | Medium | |
| Ren (2009) | Y | Y | Y | Y | Y | Y | U | Y | N | Y | Low | |
| Ren (2011) | Y | Y | Y | Y | Y | Y | Y | Y | Y | Y | Low | |
| Sentilhes (2013) | Y | Y | Y | Y | Y | Y | Y | Y | Y | Y | Low | |
| Seo (2014) | Y | Y | Y | Y | Y | Y | Y | Y | Y | Y | Low | |
| Shah (2022) | Y | Y | Y | Y | Y | Y | Y | Y | Y | Y | Low | |
| Shan (2019) | Y | Y | Y | Y | Y | Y | Y | Y | Y | U | Low | |
| Shi (2023) | Y | Y | Y | Y | Y | Y | N | Y | Y | Y | Low | |
| Singleton (2010) | Y | Y | Y | Y | Y | Y | Y | Y | Y | Y | Low | |
| Sipulwa (2016) | Y | Y | Y | Y | Y | Y | Y | Y | Y | Y | Low | |
| Sonawane (2019) | Y | Y | Y | Y | Y | Y | Y | Y | Y | Y | Low | |
| Sonmezer (2023) | Y | Y | Y | Y | Y | N | Y | Y | N | Y | Low | |
| Srinivasan (2013) | Y | Y | Y | Y | Y | Y | Y | Y | Y | Y | Low | |
| Sung (2009) | Y | Y | Y | Y | Y | N | N | Y | Y | Y | Low | |
| Suzuki (2012) | Y | Y | Y | Y | Y | Y | Y | Y | Y | Y | Low | |
| Tao (2018) | Y | Y | Y | Y | Y | Y | Y | Y | Y | Y | Low | |
| Thaemboonlers (2005) | N | Y | U | Y | Y | Y | Y | Y | Y | Y | Low | |
| Trometta (2016) | Y | Y | Y | Y | Y | Y | Y | Y | Y | Y | Low | |
| Tsagarakis (2017) | N | Y | U | Y | Y | U | Y | Y | Y | Y | Medium | |
| Tuz (2022) | Y | Y | Y | Y | Y | Y | Y | Y | Y | Y | Low | |
| Vabret (2008) | Y | Y | Y | Y | Y | Y | Y | Y | Y | Y | Low | |
| Van Elden (2004) | Y | Y | Y | Y | Y | N | N | Y | N | Y | Medium | |
| Varghese (2018) | Y | Y | Y | Y | Y | Y | Y | Y | Y | Y | Low | |
| Venter (2011) | Y | Y | Y | Y | Y | Y | Y | Y | Y | Y | Low | |
| Visseaux (2017) | U | Y | Y | Y | Y | U | N | Y | U | Y | Medium | |
| Wansaula (2015) | Y | Y | Y | Y | Y | Y | Y | Y | Y | Y | Low | |
| Wertheim (2015) | Y | Y | Y | Y | Y | N | N | Y | N | Y | Medium | |
| Wong-Chew (2017) | Y | Y | Y | Y | Y | N | Y | Y | Y | Y | Low | |
| Woo (2012) | N | U | U | Y | Y | Y | Y | Y | Y | Y | Medium | |
| Xin (2012) | U | Y | Y | Y | U | Y | Y | Y | Y | Y | Low | |
| Xu (2021) | Y | Y | Y | Y | Y | Y | Y | Y | Y | Y | Low | |
| Ye (2017) | Y | Y | Y | Y | Y | Y | Y | Y | Y | Y | Low | |
| Ye (2023) | Y | Y | Y | Y | Y | Y | Y | Y | Y | Y | Low | |
| Yew (2019) | Y | Y | Y | Y | Y | Y | Y | Y | Y | Y | Low | |
| Yip (2016) | Y | Y | Y | Y | Y | Y | N | Y | U | Y | Low | |
| Yu (2012) | Y | Y | Y | Y | Y | U | Y | Y | Y | Y | Low | |
| Zeng (2018) | Y | Y | Y | Y | Y | Y | Y | Y | Y | Y | Low | |
| Zhang (2012) | Y | Y | Y | Y | Y | Y | Y | Y | Y | Y | Low | |
| Zhang (2021) | Y | Y | Y | Y | Y | Y | Y | Y | Y | Y | Low | |
| Zhang, D (2013) | Y | Y | Y | Y | Y | N | N | Y | N | Y | Medium | |
| Zhang, S-F (2018) | Y | Y | Y | Y | Y | Y | Y | Y | Y | Y | Low | |
| Zhao (2019) | Y | Y | Y | Y | Y | Y | Y | Y | Y | Y | Low | |
| Zhu (2021) | Y | Y | Y | Y | Y | Y | Y | Y | U | Y | Low | |

### **Cohort studies**

|  | 1. Were the two groups similar and recruited from the same population? | 2. Were the exposures measured similarly to assign people to both exposed and unexposed groups? | 3. Was the exposure measured in a valid and reliable way? | 4. Were confounding factors identified? | 5. Were strategies to deal with confounding factors stated? | 6. Were the groups/participants free of the outcome at the start of the study (or at the moment of exposure)? | 7. Were the outcomes measured in a valid and reliable way? | 8. Was the follow up time reported and sufficient to be long enough for outcomes to occur? | 9. Was follow up complete, and if not, were the reasons to loss to follow up described and explored? | 10. Were strategies to address incomplete follow up utilized? | 11. Was appropriate statistical analysis used? |  | |
| --- | --- | --- | --- | --- | --- | --- | --- | --- | --- | --- | --- | --- | --- |
| Ali (2016) | Y | Y | Y | Y | Y | Y | Y | Y | Y | U | Y | Low | |
| Azziz-Baumgartner (2021) | N | U | Y | Y | Y | Y | Y | Y | Y | U | Y | Medium | |
| Cebey-Lopez (2015) | Y | Y | Y | Y | Y | Y | Y | Y | Y | U | Y | Low | |
| Fairchok (2010) | Y | Y | Y | Y | Y | Y | Y | Y | Y | Y | Y | Low | |
| Garbino (2009) | Y | Y | Y | Y | Y | Y | Y | Y | Y | Y | Y | Low | |
| Hautala (2020) | Y | Y | Y | Y | Y | Y | Y | N | N | N | Y | High | |
| Heimdal (2019) | Y | Y | Y | Y | N | Y | Y | Y | Y | U | Y | Low | |
| Kumar (2017) | Y | Y | Y | Y | Y | Y | Y | Y | Y | Y | Y | Low | |
| Kumar (2020) | Y | Y | Y | U | U | Y | Y | Y | Y | N | Y | Medium | |
| Monto (2020) | Y | Y | Y | Y | Y | Y | Y | Y | Y | Y | Y | Low | |
| Razuri (2010) | Y | Y | Y | N | Y | Y | Y | Y | Y | Y | Y | Low | |
| Regamey (2008) | Y | Y | Y | Y | Y | Y | Y | Y | Y | Y | Y | Low | |
| Sarna (2017) | Y | Y | Y | Y | Y | Y | Y | Y | Y | Y | Y | Low | |
| Talbot, HK (2009a) | Y | Y | Y | Y | Y | Y | Y | Y | Y | Y | Y | Low | |
| Talbot, HK (2009b) | Y | U | Y | U | U | Y | Y | Y | Y | Y | Y | Low | |
| Uddin (2018) | Y | Y | Y | Y | Y | Y | Y | Y | Y | Y | Y | Low |  |
| van der Zalm (2009) | Y | Y | Y | Y | Y | Y | Y | Y | Y | Y | Y | Low |  |

### **Case-control studies**

|  | 1. Were the groups comparable other than the presence of disease in cases or the absence of disease in controls? | 2. Were cases and controls matched appropriately? | 3. Were the same criteria used for identification of cases and controls? | 4. Was exposure measured in a standard, valid and reliable way? | 5. Was exposure measured in the same way for cases and controls? | 6. Were confounding factors identified? | 7. Were strategies to deal with confounding factors stated? | 8. Were outcomes assessed in a standard, valid and reliable way for cases and controls? | 9. Was the exposure period of interest long enough to be meaningful? | 10. Was appropriate statistical analysis used? |  |
| --- | --- | --- | --- | --- | --- | --- | --- | --- | --- | --- | --- |
| Baillie (2021) | Y | Y | Y | Y | Y | Y | Y | Y | U | Y | Low |
| Berkley (2010) | Y | Y | Y | Y | Y | Y | Y | Y | Y | Y | Low |
| Dare (2007) | Y | Y | Y | Y | Y | N | U | U | Y | Y | Medium |
| Jean (2013) | Y | Y | Y | Y | Y | Y | U | Y | Y | Y | Low |
| Leven (2018) | Y | Y | Y | Y | Y | Y | Y | Y | Y | Y | Low |
| Nicol (2021) | Y | Y | U | Y | Y | Y | Y | Y | Y | Y | Low |
| Owusu (2014) | Y | N | Y | Y | Y | U | U | Y | Y | Y | Medium |
| Rhedin (2015) | Y | Y | Y | Y | Y | Y | Y | Y | Y | Y | Low |
| van Gageldonk-Lafeber (2005) | Y | Y | Y | Y | Y | Y | Y | Y | Y | Y | Low |
